# PathLocdb: a comprehensive database for the subcellular localization of metabolic pathways and its application to multiple localization analysis

**DOI:** 10.1186/1471-2164-11-S4-S13

**Published:** 2010-12-02

**Authors:** Min Zhao, Hong Qu

**Affiliations:** 1 Center for Bioinformatics, National Laboratory of Protein Engineering and Plant Genetic Engineering, College of Life Sciences, Peking University, Beijing, 100871, P.R. China; 2 Key Laboratory of Evolutionary Systematics of Vertebrates, Institute of Vertebrate Paleontology and Paleoanthropology, Chinese Academy of Sciences, Beijing, 100044, China

## Abstract

**Background:**

In eukaryotes, the cell is divided into several compartments enclosed by unitary membranes. Such compartmentalization is critical for cells to restrict different pathways to be carried out in different subcellular regions. The summary and classification of subcellular localizations of metabolic pathways are the first steps towards understanding their roles in spatial differentiation and the specialization of metabolic pathways in different organisms.

**Results:**

Integrating the subcellular localization of enzymes and their pathways from UniProt Knowledgebase and KEGG pathway databases, we present the first database for subcellular localization of 43014 pathways from 80676 UniProt entries and their pathway annotations from UniProt and KEGG pathway databases. To extract pathway localization across organisms, we defined 889 superpathways as clusters of basic pathways with the same pathway annotations from different organisms. Over eighty-eight percent of superpathways in the Swiss-Prot dataset occur in cytoplasm and mitochondria. And over seventy percent of UniProt superpathways have multiple localization annotations. We summarized four common reasons for the multiple localization of superpathways. Based on this database, we also discovered 88 potential transport systems between different steps of multiply localized pathways and 45 duplicated genes from 17 pathways, occurring in parallel in several locations in humans.

**Conclusions:**

PathLocdb is a free web-accessible database that enables biochemical researchers to quickly access summarized subcellular localization of pathways from UniProt and KEGG pathway databases. As the first effort to systematically integrate pathway localization, this database is very useful in discovering the variation of localization of pathways between organisms and also cross-talk between different organelles within a pathway. The Pathlocdb database is available at http://pathloc.cbi.pku.edu.cn.

## Background

In eukaryotes, cells are subdivided into membrane-bound subcellular organelles [1]. Subcellular localization of metabolic enzymes can give precise control over where these are synthesized and operate [2]. Such compartmentalization of metabolic enzymes and metabolites of pathways provides a regulatory mechanism to control metabolic pathways. Moreover, many pathways such as the β-oxidation of fatty acids occur in several subcellular organelles. The differentiation of localization of these pathways often causes the differences in their efficiency in utilizing the metabolites [3,4]. The regulatory mechanisms for coordinating the different metabolic environments in different organelles are more complex [1]. Furthermore, incorrect localization of enzymes are often implicated in serious diseases [5]. Thus, understanding the distribution patterns of pathway localization is essential in discovering potential regulatory mechanisms and the localization of metabolic pathways. In addition, the spatial distribution pattern information of pathways is also important in clarifying pathway boundaries and in discovering the mechanism of intermediate communication between different subcellular compartments [6].

However, the subcellular localization information of pathways and their related enzymes has not been systematically integrated. The popular pathway databases such as KEGG, BioCyc, MPW and aMAZE are constructed for specific research tasks such as the graphic representation or reconstruction of metabolic pathways [7-10]. Thus, they seldom provide integrated localization information.

Summary and classification of subcellular localization of metabolic pathways are the first steps towards understanding their roles in spatial differentiation and functional specialization. Here, we present the first effort to systematically collect pathway localization information from the UniProt [11] and KEGG Ligand databases [7]. The strategy to summarize pathway localization is mainly based on the pathway and subcellular localization annotations of their participating enzymes. Using an automatic pipeline, the subcellular localization data of 43014 pathways were integrated from 80676 UniProt entries and their pathway annotations from UniProt and KEGG pathway databases. Furthermore, 889 superpathways involving 33953 organisms were summarized from the 43014 pathways. Our results indicate that over eighty-eight percent of superpathways in the UniProt dataset occur in the cytoplasm and the mitochondria. Also, over 70% of UniProt superpathways contain multiple locations. From our data, we have proposed four common reasons for multiple localization of superpathways. As the first system-wide collection for the subcellular localization of metabolic pathways, PathLocdb provides a valuable understanding of distribution patterns of metabolic pathways among cellular organelles in different organisms.

## Results

Integration of UniProt and KEGG Ligand databases provides many clues for studying pathways and pathway evolution. Here we present four types of applications for further studies.

### Differences in data content and pathway localization annotations from UniProt and KEGG databases

Our strategy to summarize the pathway localization was mainly based on the pathway and subcellular localization annotations from their participating enzymes. Thus, only enzymes with both subcellular localization and pathway annotations satisfied our criterion. The enzyme localization data are from UniProtKB/Swiss-Prot (version 56.8) in UniProt (version 14.8, Feb. 2009) databases. The pathway annotations were fro UniProtKB/Swiss-Prot (version 56.8) and KEGG ligand databases (version 44.0). In total, the Pathlocdb database consists of subcellular localization information of 43014 pathways summarized from 80676 UniProt entries and their pathway annotations from UniProt and KEGG pathway databases. To extract pathway localization across organisms, we defined a superpathway as a cluster of basic pathways with the same name from different organisms. A total of 889 superpathways across 33953 organisms were extracted from 43014 pathways. Due to differences in data quality and in pathway annotations, three datasets were collected. The high quality dataset contains 3448 pathways with localization information as well as 337 annotated superpathways across 795 organisms summarized from 6630 UniProtKB/Swiss-Prot entries. The KEGG pathway localization dataset includes 2535 pathways and 215 superpathways with localization information of the participating enzymes summarized from 12281 UniProtKB/Swiss-Prot entries of 42 organisms. A comprehensive dataset including 37029 pathways with localization information was collected from 70566 UniProt entries involving 33953 organisms.

**Figure 1:**
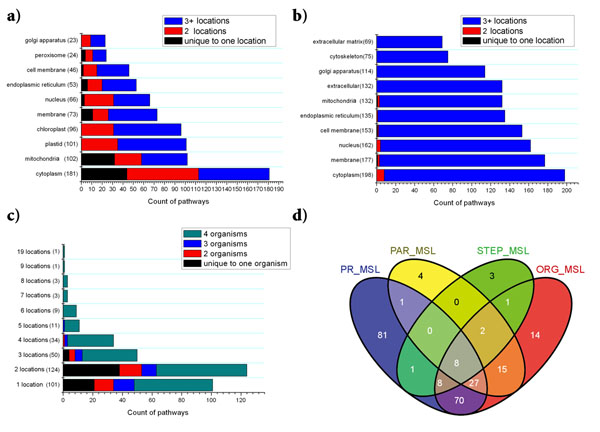
**Data contents of PathLocdb and the classification of multiple localizing superpathways.** (a, b) Distribution of 337 Swiss-Prot superpathways and 215 KEGG superpathways in their top ten organelles. For each bar, superpathways unique to the subcellular locations on Y axis are represented in black; the pathways with two or more than three locations including that on the Y axis are shown in red and blue respectively, (c) Distribution of 337 Swiss-Prot superpathways with different numbers of localizations. For each bar, superpathways specific to single organism are represented in black; the pathways with two, three or more than four organisms including that on the Y axis are shown in red, blue and navy respectively, (d) Venn diagram for the multiple locations of 235 Swiss-Prot superpathways. The ORG_MSL represents the superpathways with multiple locations from different organisms; The STEP_MSL represents pathways carried out through a series of steps spanning several subcellular locations; the PAR_MSL shows pathways occurring parallel in several subcellular locations ; and the PR_MSL represents the count of pathways with multiple localizing enzymes.

A quick comparison of localization distribution of superpathways in UniProtKB/Swiss-Prot and KEGG datasets reveals different pathway notations. The top ten subcellular locations where superpathways localize in UniProtKB/Swiss-Prot dataset cover more than 99% of annotated superpathways (Fig. 1a). Although the top ten organelle locations in KEGG dataset also have a high coverage of all annotated superpathways, organelle locations and their coverage are different from those of the UniProtKB/ Swiss-Prot dataset (Fig. 1b). Over 88% of superpathways in the UniProtKB/Swiss-Prot dataset occur in cytoplasm and mitochondria, whereas more nuclear and cell membrane superpathways are summarized in the KEGG dataset. On average, as the KEGG pathways usually combine multiple pathways from different organisms, each one of them spans over 8 locations. By contrast, most of UniProtKB/Swiss-Prot superpathways are localized in less than four organelles (Fig. 1c). Regarding the organisms involved, 63 of 337 UniProtKB/Swiss-Prot superpathways occur in single organisms while the remaining 274 superpathways occur in multiple organisms. Also from the view of pathway localization, our comparison of superpathway localization clearly reveals that UniProtKB/Swiss-Prot pathways are smaller in size than pathways from the KEGG dataset. Thus caution should be taken when interpreting pathway localization from different pathway databases.

### Discovery of conserved pathway locations across organisms and organism specific pathway localization

As the pathways in UniProtKB/Swiss-Prot do not combine biological processes of different organisms, it is useful to survey pathway localization differences in different organisms. Many fundamental biological processes with conserved localization are functionally related to diseases. For instance, the pathway “polyol metabolism; myo-inositol degradation into D-glucuronate; D-glucuronate from myo-inositol” in our database is involved in the phosphatidylinositol second messenger system (PI-cycle). The change of its conserved location in the cytoplasm to other regions could cause many neurological diseases [5]. The simplest conserved pathway localization pattern is the 85 superpathways with one single conserved location across several organisms in our database. We also find nine superpathways across more than 17 organisms satisfying the simplest conservation criterion of one single location: five in cytoplasm, three in mitochondria and one in peroxisome (Table 1). Consistent with previous studies [12], the three fundamental pathways in mitochondria have highly conserved localization. “Oxidative phosphorylation” localizes to the mitochondria in 33283 organisms in the UniProt dataset, making it the most conserved localization of superpathway in our database.

**Table 1:** Nine superpathways with one single conserved location across organisms.

*Supepathway*	*Location*	*Organisms*
Amino-acid biosynthesis ; S-adenosyl-L-methionine biosynthesis ; S-adenosyl-L-methionine from L-methionine	cytoplasm	44
Carbohydrate degradation ; glycolysis ; D-glyceraldehyde 3-phosphate from glycerone phosphate	cytoplasm	16
Cofactor biosynthesis; ubiquinone biosynthesis	mitochondria	21
Energy metabolism; oxidative phosphorylation	mitochondria	178
Fermentation ; pyruvate fermentation to lactate ; (S) -lactate from pyruvate	cytoplasm	59
Lipid metabolism; peroxisomal fatty acid beta-oxidation	peroxisome	18
Phenylpropanoid metabolism ; cinnamic acid biosynthesis ; trans-cinnamic acid from L-phenylalanine	cytoplasm	36
Porphyrin metabolism ; protoporphyrin-IX biosynthesis ; 5-aminolevulinate from glycine	mitochondria	20
Purine metabolism ; GMP biosynthesis ; GMP from XMP (glutamine route)	cytoplasm	18

The Organism row is the count of all the organisms with single conserved superpathway localization.

Using our database, 63 organism specific superpathways are found (Fig. 1c), 35 of them being plant-specific such as “abscisic acid (ABA) biosynthesis” [13], and 21 being fungi-specific. Pathway localization specific to certain organisms could also be found by sorting out completely different patterns of localization between different organisms. For instance, the gene *Cullin-1* in our database belonging to “protein modification; protein neddylation” in *Arabidopsis thaliana* mainly localizes to the nucleus during interphase and preprophase [14]. This is obviously different from homologous genes in mammals with their cellular membrane and lipid raft localization [15]. In total, 56 superpathways with such completely different localization patterns across organisms have been discovered (Additional file 1). Moreover, 184 superpathways with partially different localization patterns between organisms (Additional file 2) have also been found. For example, “glycan biosynthesis; starch biosynthesis” in all surveyed plants are carried out in the chloroplast and the plastid, but the superpathways from some plants could localize additionally to the amyloplast.

### Classification of multiple subcellular locations of metabolic pathways and prediction of potential transport systems

Pathways with multiple locations are a prevalent phenomenon. Among the annotated 337 UniProtKB/Swiss-Prot superpathways, 235 of them occur in multiple locations. Here we propose four common possible reasons giving rise to these multiple localization annotation of 337 UniProtKB/Swiss-Prot superpathways at the subcellular level. Firstly, different organisms have different pathways, resulting in multiple annotations of subcellular localization of superpathways. The most common examples for this category are the pathways which generate ATP from mitochondria and chloroplasts in different organism groups. Secondly, many pathways are composed of a series of steps spanning several subcellular locations. Like a production line, many intermetabolites are produced from one organelle to another, stepwise in pathways. Thirdly, some pathways occur in parallel in several subcellular locations in a single organism. The obvious example is fatty acid β-oxidation occurring in the mitochondria as well as the peroxisome [1]. The other most common reason for multiple annotation of pathways is the fact that they are summarized from multiple localizing enzymes. An extreme example here is the first enzyme of GPI biosysthesis in humans, with five subunits [16-18]. Four of its five subunits localize in the endoplasmic reticulum and the remaining one in the cytoplasm. However, the above four phenomena are often mixed together when we try to analyze the reasons for multiple localization of pathways (Fig. 1d).

Regardless of the complex reasons leading to multiple annotation of pathway localization, each possible reason is useful for researchers to further explore its potential significance. The transport system or cross-talk between organelles are made up of common processes for translocation of intermediates [6,19]. Focusing on the 3448 pathways of the UniProtKB/Swiss-Prot dataset, 88 pathways with multiple locations are summarized from the multiple localizations of their translocation steps (Additional file 3). These results provide information on the potential existence of a transport system between these pathways with different locations and different steps. For instance, the pathway titled “amine and polyamine degradation; betaine degradation; sarcosine from betaine” consists of two steps in human, mouse and rat. The first step of this pathway occurs in cytoplasm in the three organisms, while the second step is in the mitochondria. Further experimental studies on the potential transport system between the first and second steps of the pathway in mammals may be interesting and useful.

### Pathways occurring in parallel in multiple locations and duplicated pathways in human

Using our database, 28 pathways occurring in parallel in several locations in human are found. On combini ng these pathways with the gene duplication dataset in human [20,21], 45 genes from 17 of the 28 pathways are detected to be duplicated during evolution (Additional file 4). Taking two duplicated genes *17-beta-HSD 1* and *17-beta-HSD 12* as examples, their protein products are localized to the cytoplasm and the endoplasmic reticulum respectively. Both proteins are involved in the “Steroid biosynthesis; estrogen biosynthesis” pathway. Similar phenomena also exist in the “Protein modification; protein sumoylation” and “Protein modification; protein ubiquitination” pathways in human. Occurrence of such a high proportion of enzyme duplication during pathway evolution also deserves further study.

## Discussion

To our knowledge, the present study represents the first of its kind, presenting a genome-wide collection of subcellular localization of metabolic pathways in cells across 33953 organisms. With high quality data from the UniProtKB/Swiss-Prot and KEGG databases, we have compared the pathway localization annotations of the two databases and found that the contributions from KEGG are larger than those from UniProtKB/Swiss-Prot.

A characteristic feature of this study is the summary of common reasons for the multiple annotations of pathway localization. Recently high throughput proteomics data suggest that proteins with multiple locations constitute 39 percent of the total 1404 proteins in mouse liver [22]. Our database shows that over 70% of UniProtKB/Swiss-Prot superpathways have multiple localization annotations. The consistent phenomena at the pathway level confirm that differentiation of localization of biological function is prevalent. Intensive studies on the pathways which are carried out through a series of steps spanning several subcellular locations reveal 88 potential transport systems between different steps of multiple localizing pathways.

Based on the multiple localization pathways from different organisms, we discovered possibly for the first time, all the conserved pathway localizations across organisms and organism-specific pathway localizations. This systematic comparison of pathway localization between organisms reveals that numerous pathways occur at different locations, providing clues for the differentiation and specialization of the pathway localizations between organisms.

Focusing on the 28 pathways occurring in parallel in multiple locations in human, we identified 17 pathways containing gene duplication events to gain new locations. According to one of the most popular pathway evolution models, the “patchwork model”, enzymes with broad substrate specificities are more likely to be recruited to execute a new metabolic function [23]. Although our analyses show that a high proportion of enzyme duplication occurs during pathway evolution, the mechanism of gaining new locations need to be elucidated. Phylogenetic distribution of N-terminal targeting signals of mitochondrial localizing proteins suggest that inter-compartmental duplication events could bring novel localization of gene products and expand the catalytic as well as the RNA processing repertoire [24]. While multiple localizations of metabolic pathways provide more valuable clues for researchers to discover potential evolution routes, we hope further study will elucidate the possible mechanisms.

In conclusion, these analyses demonstrate that our database is valuable in the discovery of potential cross-talk between different steps and the variation of localization of pathways between organisms.

## Conclusions

PathLocdb was constructed as a free web-accessible database and analysis server to enable biochemical researchers to quickly access to summarized subcellular localization of metabolic pathways of UniProt and KEGG pathway database. As the first effort to systematically collect pathway localization, this database is very useful in discovering the variation of localization of pathways between organisms and also cross-talk between different organelles within a pathway. The PathLocdb database can be accessed at http://pathloc.cbi.pku.edu.cn.

## Methods

### Data source and automation of data collection

An automatic computational pipeline was implemented to extract subcellular localization and pathway annotations from UniProtKB/Swiss-Prot and KEGG ligand databases using Perl scripts and the Swissknife module[25]. Eukaryotic protein entries were screened by its taxonomy identification within the taxonomic groups including fungi, invertebrates, vertebrates, plants, mammals, rodents and human. As our investigation was focused on the cellular organelle level, all the subcellular localization descriptions were formatted at the organelle level. For instance, the “Mitochondrial intermembrane space” is assigned to Mitochondria. All the subcellular localization descriptions marked with “By similarity”, “Probable” and “Potential” were also accepted. In UniProtKB/Swiss-Prot, pathway annotations are in free text and step information was extracted using keywords, such as “step 2/2”.

To compare the subcellular localization annotations of pathways from different organisms, we defined a superpathway as a cluster of pathways having the same annotation from different organisms. The reference pathways in KEGG are combined multiple pathways from different organisms[8]. Thus, the superpathway name of the KEGG dataset is the same as the name of reference pathways. As the pathway names from UniProt are also formatted to structured controlled vocabulary in UniPathway [26], the superpathway name of the UniProtKB/Swiss-Prot dataset is the same as that in UniPathway.

As an automatic pipeline for data collection, our database will be easy to update regularly when new versions of Swiss-Prot and KEGG Ligand are available. Besides, more data from other pathway databases and literatures will be compiled in the future.

### Web interface construction

**Figure 2:**
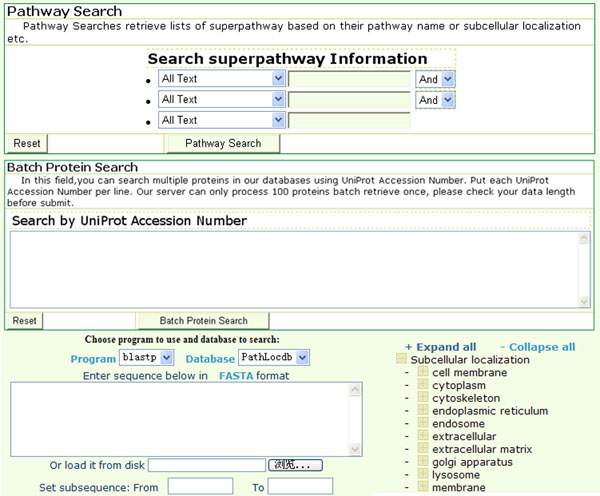
**Web interface of PathLocdb.** There are four functions for users to obtain data: key word query, batch accession number download, Blast search against all the proteins in PathLocdb and also browse all the data from different features such as subcellular localization. By typing accession numbers, users can get the multiple sequences in PathLocdb at one retrieve. And in browser menu, users could browse data hierarchically.

All data and information in PathLocdb were stored in a MySQL relational database on a Linux server. The database provides a web interface that allows researchers to hierarchically browse the subcellular localizations of 887 superpathways using different features (Fig. 2). Web-based queries to the database were implemented in Perl scripts running in an Apache environment. PathLocdb allows users to query by enzyme or gene, to browse by KEGG pathway maps, subcellular localization, or to run BLAST searches against the sequences in PathLocdb. For an advanced study, this database provides all protein sequences in FASTA format. A feedback page was implemented to collect comments on existing records or suggestions of new pathway localization from users.

## Authors’ contributions

MZ conceived of the work, carried out all analyses and helped write the manuscript. HQ helped write the manuscript.

## Supplementary Material

Additional file 1 The summarized 56 superpathways with completely different locations across organisms are shown in Additional file 1. Click here for file

Additional file 2 The 184 superpathways with partially different localization patterns between organisms are shown in Additional file 2. Click here for file

Additional file 3 The 88 pathways with multiple locations summarized from the multiple locations of their steps are shown in Additional file 3. They provide information on potential existence of transport system between these pathways with different localizations and different steps. Click here for file

Additional file 4 The 45 genes from 17 of the 28 pathways which are detected to be duplicated during evolution are shown in Additional file 4. Click here for file
