# Supplementary material for: PathLocdb: a comprehensive database for the subcellular localization of metabolic pathways and its application to multiple localization analysis
Source: BMC Genomics. 2010 Dec 2;11(Suppl 4):S13. doi: 10.1186/1471-2164-11-S4-S13 (PMC3005916; doi:10.1186/1471-2164-11-S4-S13)
Supplement: Additional file 1 — The summarized 56 superpathways with completely different locations across organisms are shown in Additional file 1. [file 1471-2164-11-S4-S13-S1.pdf]

PathLoc superpathway ID	Superpathways with completely different locations between organisms
PLSP10	Amine and polyamine biosynthesis ; betaine biosynthesis via choline pathway ; betaine from betaine aldehyde
PLSP19	Amino-acid biosynthesis ; L-arginine biosynthesis ; L-arginine from L-ornithine and carbamoyl phosphate
PLSP21	Amino-acid biosynthesis ; L-arginine biosynthesis ; N(2)-acetyl-L-ornithine from L-glutamate
PLSP21	Amino-acid biosynthesis ; L-arginine biosynthesis ; N(2)-acetyl-L-ornithine from L-glutamate
PLSP22	Amino-acid biosynthesis ; L-arginine biosynthesis ; carbamoyl phosphate from HCO(3)(-)
PLSP27	Amino-acid biosynthesis ; L-histidine biosynthesis ; L-histidine from 5-phospho-alpha-D-ribose 1-diphosphate
PLSP27	Amino-acid biosynthesis ; L-histidine biosynthesis ; L-histidine from 5-phospho-alpha-D-ribose 1-diphosphate
PLSP27	Amino-acid biosynthesis ; L-histidine biosynthesis ; L-histidine from 5-phospho-alpha-D-ribose 1-diphosphate
PLSP29	Amino-acid biosynthesis ; L-isoleucine biosynthesis ; L-isoleucine from 2-oxobutanoate
PLSP30	Amino-acid biosynthesis ; L-leucine biosynthesis ; L-leucine from 3-methyl-2-oxobutanoate
PLSP30	Amino-acid biosynthesis ; L-leucine biosynthesis ; L-leucine from 3-methyl-2-oxobutanoate
PLSP31	Amino-acid biosynthesis ; L-lysine biosynthesis via AAA pathway ; L-alpha-aminoadipate from 2-oxoglutarate
PLSP44	Amino-acid biosynthesis ; L-proline biosynthesis ; L-glutamate 5-semialdehyde from L-glutamate
PLSP45	Amino-acid biosynthesis ; L-proline biosynthesis ; L-glutamate 5-semialdehyde from L-ornithine
PLSP46	Amino-acid biosynthesis ; L-proline biosynthesis ; L-proline from L-glutamate 5-semialdehyde
PLSP51	Amino-acid biosynthesis ; L-valine biosynthesis ; L-valine from pyruvate
PLSP54	Amino-acid biosynthesis; L-methionine biosynthesis via de novo pathway
PLSP62	Amino-acid degradation ; L-lysine degradation via saccharopine pathway ; glutaryl-CoA from L-lysine
PLSP64	Amino-acid degradation ; L-proline degradation into L-glutamate ; L-glutamate from L-proline
PLSP67	Amino-acid degradation; 4-aminobutanoate degradation
PLSP88	Carbohydrate degradation ; pentose phosphate pathway ; D-ribulose 5-phosphate from D-glucose 6-phosphate (oxidative stage)
PLSP92	Carbohydrate metabolism ; glyoxylate cycle ; L-malate from isocitrate

PLSP99	Carotenoid biosynthesis ; phytoene biosynthesis ; phytoene from geranylgeranyl-PP
PLSP111	Cofactor biosynthesis ; FMN biosynthesis ; FMN from riboflavin (ATP route)
PLSP129	Cofactor biosynthesis ; tetrahydrofolate biosynthesis ; 2-amino-4-hydroxy-6-hydroxymethyl-7,8-dihydropteridine diphosphate from 2-amino-4-hydroxy-6-(erythro-1,2,3-trihydroxypropyl)-dihydropteridine triphosphate
PLSP129	Cofactor biosynthesis ; tetrahydrofolate biosynthesis ; 2-amino-4-hydroxy-6-hydroxymethyl-7,8-dihydropteridine diphosphate from 2-amino-4-hydroxy-6-(erythro-1,2,3-trihydroxypropyl)-dihydropteridine triphosphate
PLSP131	Cofactor biosynthesis ; tetrahydrofolate biosynthesis ; dihydrofolate from 2-amino-4-hydroxy-6-hydroxymethyl-7,8-dihydropteridine diphosphate and 4-aminobenzoate
PLSP149	Energy metabolism; sulfur metabolism
PLSP156	Glycan biosynthesis; sucrose metabolism
PLSP159	Glycan metabolism ; pectin degradation ; 2-dehydro-3-deoxy-D-gluconic acid from pectin
PLSP176	Isoprenoid biosynthesis ; dimethylallyl-PP biosynthesis ; dimethylallyl-PP from isopentenyl-PP
PLSP209	Metabolic intermediate biosynthesis ; chorismate biosynthesis ; chorismate from D-erythrose 4-phosphate and PEP
PLSP209	Metabolic intermediate biosynthesis ; chorismate biosynthesis ; chorismate from D-erythrose 4-phosphate and PEP
PLSP209	Metabolic intermediate biosynthesis ; chorismate biosynthesis ; chorismate from D-erythrose 4-phosphate and PEP
PLSP209	Metabolic intermediate biosynthesis ; chorismate biosynthesis ; chorismate from D-erythrose 4-phosphate and PEP
PLSP209	Metabolic intermediate biosynthesis ; chorismate biosynthesis ; chorismate from D-erythrose 4-phosphate and PEP
PLSP210	Metabolic intermediate biosynthesis ; mevalonic acid biosynthesis ; (R)-mevalonic acid from acetyl-CoA
PLSP235	Phospholipid metabolism ; phosphatidylethanolamine biosynthesis ; phosphatidylethanolamine from CDP-diacylglycerol
PLSP254	Plant hormone degradation; abscisic acid degradation
PLSP269	Porphyrin metabolism ; protoporphyrin-IX biosynthesis ; coproporphyrinogen III from 5-aminolevulinate
PLSP271	Porphyrin metabolism ; protoporphyrin-IX biosynthesis ; protoporphyrinogen-IX from coproporphyrinogen III (O2 route)

PLSP278	Protein modification ; protein lipoylation via endogenous pathway ; protein N(6)-(lipoyl)lysine from octanoyl-[acyl-carrier-protein]
PLSP279	Protein modification ; protein lipoylation via exogenous pathway ; protein N(6)-(lipoyl)lysine from lipoic acid
PLSP285	Protein modification; protein neddylation
PLSP293	Purine metabolism ; IMP biosynthesis via de novo pathway ; 5-amino-1-(5-phospho-D-ribosyl)imidazole-4-carboxamide from N(2)-formyl-N(1)-(5-phospho-D-ribosyl)glycinamide
PLSP296	Purine metabolism ; IMP biosynthesis via salvage pathway ; IMP from AMP
PLSP301	Purine metabolism ; uric acid degradation ; (S)-allantoin from uric acid
PLSP301	Purine metabolism ; uric acid degradation ; (S)-allantoin from uric acid
PLSP303	Pyrimidine metabolism ; UMP biosynthesis via de novo pathway ; UMP from HCO(3)(-)
PLSP303	Pyrimidine metabolism ; UMP biosynthesis via de novo pathway ; UMP from HCO(3)(-)
PLSP303	Pyrimidine metabolism ; UMP biosynthesis via de novo pathway ; UMP from HCO(3)(-)
PLSP304	Pyrimidine metabolism ; UMP biosynthesis via salvage pathway ; UMP from uracil
PLSP318	Steroid biosynthesis ; zymosterol biosynthesis ; zymosterol from lanosterol
PLSP318	Steroid biosynthesis ; zymosterol biosynthesis ; zymosterol from lanosterol
PLSP326	Sulfur metabolism ; glutathione biosynthesis ; glutathione from L-cysteine and L-glutamate
PLSP332	Terpene metabolism ; lanosterol biosynthesis ; lanosterol from farnesyl-PP
