# Supplementary material for: PathLocdb: a comprehensive database for the subcellular localization of metabolic pathways and its application to multiple localization analysis
Source: BMC Genomics. 2010 Dec 2;11(Suppl 4):S13. doi: 10.1186/1471-2164-11-S4-S13 (PMC3005916; doi:10.1186/1471-2164-11-S4-S13)
Supplement: Additional file 2 — The 184 superpathways with partially different localization patterns between organisms are shown in Additional file 2. [file 1471-2164-11-S4-S13-S2.pdf]

PathLoc superpathway ID	Superpathways with partially different locations between organisms
PLSP1	Alcohol metabolism ; ethanol degradation ; acetate from ethanol
PLSP10	Amine and polyamine biosynthesis ; betaine biosynthesis via choline pathway ; betaine from betaine aldehyde
PLSP11	Amine and polyamine biosynthesis ; creatine biosynthesis ; creatine from L-arginine and glycine
PLSP13	Amine and polyamine biosynthesis; carnitine biosynthesis
PLSP17	Amine and polyamine degradation; spermine degradation
PLSP19	Amino-acid biosynthesis ; L-arginine biosynthesis ; L-arginine from L-ornithine and carbamoyl phosphate
PLSP21	Amino-acid biosynthesis ; L-arginine biosynthesis ; N(2)-acetyl-L-ornithine from L-glutamate
PLSP21	Amino-acid biosynthesis ; L-arginine biosynthesis ; N(2)-acetyl-L-ornithine from L-glutamate
PLSP22	Amino-acid biosynthesis ; L-arginine biosynthesis ; carbamoyl phosphate from HCO(3)(-)
PLSP24	Amino-acid biosynthesis ; L-cysteine biosynthesis ; L-cysteine from L-serine
PLSP24	Amino-acid biosynthesis ; L-cysteine biosynthesis ; L-cysteine from L-serine
PLSP27	Amino-acid biosynthesis ; L-histidine biosynthesis ; L-histidine from 5-phospho-alpha-D-ribose 1-diphosphate
PLSP27	Amino-acid biosynthesis ; L-histidine biosynthesis ; L-histidine from 5-phospho-alpha-D-ribose 1-diphosphate
PLSP27	Amino-acid biosynthesis ; L-histidine biosynthesis ; L-histidine from 5-phospho-alpha-D-ribose 1-diphosphate
PLSP27	Amino-acid biosynthesis ; L-histidine biosynthesis ; L-histidine from 5-phospho-alpha-D-ribose 1-diphosphate
PLSP28	Amino-acid biosynthesis ; L-isoleucine biosynthesis ; 2-oxobutanoate from L-threonine
PLSP29	Amino-acid biosynthesis ; L-isoleucine biosynthesis ; L-isoleucine from 2-oxobutanoate
PLSP29	Amino-acid biosynthesis ; L-isoleucine biosynthesis ; L-isoleucine from 2-oxobutanoate
PLSP30	Amino-acid biosynthesis ; L-leucine biosynthesis ; L-leucine from 3-methyl-2-oxobutanoate
PLSP30	Amino-acid biosynthesis ; L-leucine biosynthesis ; L-leucine from 3-methyl-2-oxobutanoate
PLSP31	Amino-acid biosynthesis ; L-lysine biosynthesis via AAA pathway ; L-alpha-aminoadipate from 2-oxoglutarate
PLSP31	Amino-acid biosynthesis ; L-lysine biosynthesis via AAA pathway ; L-alpha-aminoadipate from 2-oxoglutarate
PLSP37	Amino-acid biosynthesis ; L-methionine biosynthesis via de novo pathway ; L-homocysteine from L-cystathionine
PLSP43	Amino-acid biosynthesis ; L-phenylalanine biosynthesis ; phenylpyruvate from prephenate

PLSP44	Amino-acid biosynthesis ; L-proline biosynthesis ; L-glutamate 5-semialdehyde from L-glutamate
PLSP45	Amino-acid biosynthesis ; L-proline biosynthesis ; L-glutamate 5-semialdehyde from L-ornithine
PLSP46	Amino-acid biosynthesis ; L-proline biosynthesis ; L-proline from L-glutamate 5-semialdehyde
PLSP49	Amino-acid biosynthesis ; L-tryptophan biosynthesis ; L-tryptophan from chorismate
PLSP51	Amino-acid biosynthesis ; L-valine biosynthesis ; L-valine from pyruvate
PLSP51	Amino-acid biosynthesis ; L-valine biosynthesis ; L-valine from pyruvate
PLSP53	Amino-acid biosynthesis ; homocysteine biosynthesis ; L-homocysteine from S-adenosyl-L-homocysteine
PLSP54	Amino-acid biosynthesis; L-methionine biosynthesis via de novo pathway
PLSP56	Amino-acid degradation ; L-alanine degradation via transaminase pathway ; pyruvate from L-alanine
PLSP58	Amino-acid degradation ; L-kynurenine degradation ; L-alanine and anthranilate from L-kynurenine
PLSP59	Amino-acid degradation ; L-kynurenine degradation ; kynurenic acid from L-kynurenine
PLSP62	Amino-acid degradation ; L-lysine degradation via saccharopine pathway ; glutaryl-CoA from L-lysine
PLSP62	Amino-acid degradation ; L-lysine degradation via saccharopine pathway ; glutaryl-CoA from L-lysine
PLSP63	Amino-acid degradation ; L-phenylalanine degradation ; acetoacetic acid and fumarate from L-phenylalanine
PLSP64	Amino-acid degradation ; L-proline degradation into L-glutamate ; L-glutamate from L-proline
PLSP67	Amino-acid degradation; 4-aminobutanoate degradation
PLSP74	Amino-sugar metabolism; N-acetylneuraminic acid metabolism
PLSP75	Aminoacyl-tRNA biosynthesis ; selenocysteinyl-tRNA(Sec) biosynthesis ; L-seryl-tRNA(Sec) from L-serine and tRNA(Sec)
PLSP81	Carbohydrate biosynthesis; Calvin cycle
PLSP82	Carbohydrate biosynthesis; gluconeogenesis
PLSP83	Carbohydrate degradation ; glycolysis ; D-glyceraldehyde 3-phosphate and glycerone phosphate from D-glucose
PLSP83	Carbohydrate degradation ; glycolysis ; D-glyceraldehyde 3-phosphate and glycerone phosphate from D-glucose
PLSP85	Carbohydrate degradation ; glycolysis ; pyruvate from D-glyceraldehyde 3-phosphate
PLSP85	Carbohydrate degradation ; glycolysis ; pyruvate from D-glyceraldehyde 3-phosphate
PLSP85	Carbohydrate degradation ; glycolysis ; pyruvate from D-glyceraldehyde 3-phosphate

PLSP85	Carbohydrate degradation ; glycolysis ; pyruvate from D-glyceraldehyde 3-phosphate
PLSP85	Carbohydrate degradation ; glycolysis ; pyruvate from D-glyceraldehyde 3-phosphate
PLSP88	Carbohydrate degradation ; pentose phosphate pathway ; D-ribulose 5-phosphate from D-glucose 6-phosphate (oxidative stage)
PLSP88	Carbohydrate degradation ; pentose phosphate pathway ; D-ribulose 5-phosphate from D-glucose 6-phosphate (oxidative stage)
PLSP92	Carbohydrate metabolism ; glyoxylate cycle ; L-malate from isocitrate
PLSP94	Carbohydrate metabolism; hexose metabolism
PLSP96	Carbohydrate metabolism; tricarboxylic acid cycle
PLSP99	Carotenoid biosynthesis ; phytoene biosynthesis ; phytoene from geranylgeranyl-PP
PLSP100	Carotenoid biosynthesis; alpha-zeacarotene biosynthesis
PLSP101	Carotenoid biosynthesis; beta-carotene biosynthesis
PLSP102	Carotenoid biosynthesis; beta-zeacarotene biosynthesis
PLSP103	Carotenoid biosynthesis; delta-carotene biosynthesis
PLSP104	Carotenoid biosynthesis; lycopene biosynthesis
PLSP111	Cofactor biosynthesis ; FMN biosynthesis ; FMN from riboflavin (ATP route)
PLSP112	Cofactor biosynthesis ; NAD(+) biosynthesis ; NAD(+) from nicotinamide ribonucleotide
PLSP113	Cofactor biosynthesis ; NAD(+) biosynthesis ; nicotinamide ribonucleotide from 5-phospho-alpha-D-ribose 1-diphosphate and nicotinamide
PLSP114	Cofactor biosynthesis ; NAD(+) biosynthesis ; nicotinate ribonucleotide from nicotinate
PLSP116	Cofactor biosynthesis ; NAD(+) biosynthesis ; pyridine-2,3-dicarboxylate from L-kynurenine
PLSP116	Cofactor biosynthesis ; NAD(+) biosynthesis ; pyridine-2,3-dicarboxylate from L-kynurenine
PLSP121	Cofactor biosynthesis ; coenzyme A biosynthesis ; coenzyme A from pantothenate
PLSP124	Cofactor biosynthesis ; pantothenate biosynthesis ; pantothenate from beta-alanine and pantoate
PLSP129	Cofactor biosynthesis ; tetrahydrofolate biosynthesis ; 2-amino-4-hydroxy-6-hydroxymethyl-7,8-dihydropteridine diphosphate from 2-amino-4-hydroxy-6-(erythro-1,2,3-trihydroxypropyl)-dihydropteridine triphosphate
PLSP129	Cofactor biosynthesis ; tetrahydrofolate biosynthesis ; 2-amino-4-hydroxy-6-hydroxymethyl-7,8-dihydropteridine diphosphate from 2-amino-4-hydroxy-6-(erythro-1,2,3-trihydroxypropyl)-dihydropteridine triphosphate

PLSP130	Cofactor biosynthesis ; tetrahydrofolate biosynthesis ; 4-aminobenzoate from chorismate
PLSP130	Cofactor biosynthesis ; tetrahydrofolate biosynthesis ; 4-aminobenzoate from chorismate
PLSP131	Cofactor biosynthesis ; tetrahydrofolate biosynthesis ; dihydrofolate from 2-amino-4-hydroxy-6-hydroxymethyl-7,8-dihydropteridine diphosphate and 4-aminobenzoate
PLSP135	Cofactor biosynthesis; molybdopterin biosynthesis
PLSP139	Cofactor biosynthesis; tetrahydrofolylpolyglutamate biosynthesis
PLSP140	Cofactor biosynthesis; thiamine pyrophosphate biosynthesis
PLSP143	Cofactor degradation ; B6 vitamer degradation ; pyridoxal from pyridoxine (dehydrogenase route)
PLSP144	Cofactor metabolism; retinol metabolism
PLSP146	Energy metabolism; nitrogen metabolism
PLSP149	Energy metabolism; sulfur metabolism
PLSP152	Genetic information processing; DNA replication
PLSP155	Glycan biosynthesis; starch biosynthesis
PLSP156	Glycan biosynthesis; sucrose metabolism
PLSP159	Glycan metabolism ; pectin degradation ; 2-dehydro-3-deoxy-D-gluconic acid from pectin
PLSP159	Glycan metabolism ; pectin degradation ; 2-dehydro-3-deoxy-D-gluconic acid from pectin
PLSP161	Glycan metabolism; N-glycan degradation
PLSP162	Glycan metabolism; N-glycan metabolism
PLSP165	Glycan metabolism; chondroitin sulfate biosynthesis
PLSP166	Glycan metabolism; heparan sulfate biosynthesis
PLSP167	Glycan metabolism; heparin biosynthesis
PLSP169	Glycerolipid metabolism; ether lipid biosynthesis
PLSP173	Glycolipid biosynthesis; glycosylphosphatidylinositol-anchor biosynthesis
PLSP176	Isoprenoid biosynthesis ; dimethylallyl-PP biosynthesis ; dimethylallyl-PP from isopentenyl-PP
PLSP177	Isoprenoid biosynthesis ; farnesyl-PP biosynthesis ; farnesyl-PP from geranyl-PP and isopentenyl-PP
PLSP178	Isoprenoid biosynthesis ; geranyl-PP biosynthesis ; geranyl-PP from dimethylallyl-PP and isopentenyl-PP
PLSP179	Isoprenoid biosynthesis ; geranylgeranyl-PP biosynthesis ; geranylgeranyl-PP from farnesyl-PP and isopentenyl-PP
PLSP180	Isoprenoid biosynthesis ; isopentenyl-PP biosynthesis via DXP pathway ; isopentenyl-PP from 1-deoxy-D-xylulose 5-phosphate
PLSP181	Isoprenoid biosynthesis ; isopentenyl-PP biosynthesis via mevalonic acid pathway ; isopentenyl-PP from (R)-mevalonic acid
PLSP181	Isoprenoid biosynthesis ; isopentenyl-PP biosynthesis via mevalonic acid pathway ; isopentenyl-PP from (R)-mevalonic acid
PLSP183	Lipid metabolism ; malonyl-CoA biosynthesis ; malonyl-CoA from acetyl-CoA

PLSP186	Lipid metabolism; bile acid biosynthesis
PLSP187	Lipid metabolism; fatty acid beta-oxidation
PLSP188	Lipid metabolism; fatty acid biosynthesis
PLSP189	Lipid metabolism; fatty acid metabolism
PLSP190	Lipid metabolism; glycerolipid metabolism
PLSP192	Lipid metabolism; leukotriene B4 biosynthesis
PLSP194	Lipid metabolism; leukotriene D4 biosynthesis
PLSP197	Lipid metabolism; oxylipin biosynthesis
PLSP199	Lipid metabolism; phospholipid metabolism
PLSP200	Lipid metabolism; polyunsaturated fatty acid biosynthesis
PLSP201	Lipid metabolism; prostaglandin biosynthesis
PLSP202	Lipid metabolism; sphingolipid metabolism
PLSP203	Lipid metabolism; steroid biosynthesis
PLSP209	Metabolic intermediate biosynthesis ; chorismate biosynthesis ; chorismate from D-erythrose 4-phosphate and PEP
PLSP209	Metabolic intermediate biosynthesis ; chorismate biosynthesis ; chorismate from D-erythrose 4-phosphate and PEP
PLSP209	Metabolic intermediate biosynthesis ; chorismate biosynthesis ; chorismate from D-erythrose 4-phosphate and PEP
PLSP209	Metabolic intermediate biosynthesis ; chorismate biosynthesis ; chorismate from D-erythrose 4-phosphate and PEP
PLSP209	Metabolic intermediate biosynthesis ; chorismate biosynthesis ; chorismate from D-erythrose 4-phosphate and PEP
PLSP210	Metabolic intermediate biosynthesis ; mevalonic acid biosynthesis ; (R)-mevalonic acid from acetyl-CoA
PLSP210	Metabolic intermediate biosynthesis ; mevalonic acid biosynthesis ; (R)-mevalonic acid from acetyl-CoA
PLSP210	Metabolic intermediate biosynthesis ; mevalonic acid biosynthesis ; (R)-mevalonic acid from acetyl-CoA
PLSP221	Nitrogen metabolism ; urea cycle ; L-ornithine and urea from L-arginine
PLSP227	One-carbon metabolism; tetrahydrofolate pathway
PLSP229	Organic acid metabolism; propionic acid degradation
PLSP231	Phospholipid metabolism ; CDP-diacylglycerol biosynthesis ; CDP-diacylglycerol from sn-glycerol 3-phosphate
PLSP231	Phospholipid metabolism ; CDP-diacylglycerol biosynthesis ; CDP-diacylglycerol from sn-glycerol 3-phosphate
PLSP231	Phospholipid metabolism ; CDP-diacylglycerol biosynthesis ; CDP-diacylglycerol from sn-glycerol 3-phosphate
PLSP232	Phospholipid metabolism ; phosphatidylcholine biosynthesis ; phosphatidylcholine from phosphocholine
PLSP232	Phospholipid metabolism ; phosphatidylcholine biosynthesis ; phosphatidylcholine from phosphocholine
PLSP235	Phospholipid metabolism ; phosphatidylethanolamine biosynthesis ; phosphatidylethanolamine from CDP-diacylglycerol
PLSP235	Phospholipid metabolism ; phosphatidylethanolamine biosynthesis ; phosphatidylethanolamine from CDP-diacylglycerol

PLSP236	Phospholipid metabolism ; phosphatidylethanolamine biosynthesis ; phosphatidylethanolamine from ethanolamine
PLSP243	Photosynthesis; C3 acid pathway
PLSP244	Photosynthesis; C4 acid pathway
PLSP251	Plant hormone biosynthesis; abscisic acid biosynthesis
PLSP253	Plant hormone biosynthesis; gibberellin biosynthesis
PLSP254	Plant hormone degradation; abscisic acid degradation
PLSP260	Polyol metabolism ; myo-inositol biosynthesis ; myo-inositol from D-glucose 6-phosphate
PLSP262	Porphyrin biosynthesis; chlorophyll biosynthesis
PLSP263	Porphyrin biosynthesis; chlorophyll biosynthesis (light-independent)
PLSP264	Porphyrin degradation; chlorophyll degradation
PLSP266	Porphyrin metabolism ; protoheme biosynthesis ; protoheme from protoporphyrin-IX
PLSP267	Porphyrin metabolism ; protoporphyrin-IX biosynthesis ; 5-aminolevulinate from L-glutamyl-tRNA(Glu)
PLSP269	Porphyrin metabolism ; protoporphyrin-IX biosynthesis ; coproporphyrinogen III from 5-aminolevulinate
PLSP269	Porphyrin metabolism ; protoporphyrin-IX biosynthesis ; coproporphyrinogen III from 5-aminolevulinate
PLSP270	Porphyrin metabolism ; protoporphyrin-IX biosynthesis ; protoporphyrin-IX from protoporphyrinogen-IX
PLSP271	Porphyrin metabolism ; protoporphyrin-IX biosynthesis ; protoporphyrinogen-IX from coproporphyrinogen III (O2 route)
PLSP276	Protein biosynthesis; polypeptide chain elongation
PLSP277	Protein degradation; proteasomal ubiquitin-dependent pathway
PLSP278	Protein modification ; protein lipoylation via endogenous pathway ; protein N(6)-(lipoyl)lysine from octanoyl-[acyl-carrier-protein]
PLSP279	Protein modification ; protein lipoylation via exogenous pathway ; protein N(6)-(lipoyl)lysine from lipoic acid
PLSP283	Protein modification; protein glycosylation
PLSP285	Protein modification; protein neddylation
PLSP286	Protein modification; protein sumoylation
PLSP287	Protein modification; protein ubiquitination
PLSP289	Purine metabolism ; AMP biosynthesis via de novo pathway ; AMP from IMP
PLSP290	Purine metabolism ; AMP biosynthesis via salvage pathway ; AMP from adenine
PLSP293	Purine metabolism ; IMP biosynthesis via de novo pathway ; 5-amino-1-(5-phospho-D-ribosyl)imidazole-4-carboxamide from N(2)-formyl-N(1)-(5-phospho-D-ribosyl)glycinamide
PLSP293	Purine metabolism ; IMP biosynthesis via de novo pathway ; 5-amino-1-(5-phospho-D-ribosyl)imidazole-4-carboxamide from N(2)-formyl-N(1)-(5-phospho-D-ribosyl)glycinamide

PLSP296	Purine metabolism ; IMP biosynthesis via salvage pathway ; IMP from AMP
PLSP297	Purine metabolism ; IMP biosynthesis via salvage pathway ; IMP from hypoxanthine
PLSP299	Purine metabolism ; cAMP degradation ; AMP from cAMP
PLSP301	Purine metabolism ; uric acid degradation ; (S)-allantoin from uric acid
PLSP301	Purine metabolism ; uric acid degradation ; (S)-allantoin from uric acid
PLSP303	Pyrimidine metabolism ; UMP biosynthesis via de novo pathway ; UMP from HCO(3)(-)
PLSP303	Pyrimidine metabolism ; UMP biosynthesis via de novo pathway ; UMP from HCO(3)(-)
PLSP303	Pyrimidine metabolism ; UMP biosynthesis via de novo pathway ; UMP from HCO(3)(-)
PLSP303	Pyrimidine metabolism ; UMP biosynthesis via de novo pathway ; UMP from HCO(3)(-)
PLSP304	Pyrimidine metabolism ; UMP biosynthesis via salvage pathway ; UMP from uracil
PLSP307	Pyrimidine metabolism ; dUMP biosynthesis ; dUMP from dCTP (dUTP route)
PLSP311	Secondary metabolite biosynthesis; flavonoid biosynthesis
PLSP313	Secondary metabolite metabolism ; methylglyoxal degradation ; D-lactate from methylglyoxal
PLSP318	Steroid biosynthesis ; zymosterol biosynthesis ; zymosterol from lanosterol
PLSP318	Steroid biosynthesis ; zymosterol biosynthesis ; zymosterol from lanosterol
PLSP318	Steroid biosynthesis ; zymosterol biosynthesis ; zymosterol from lanosterol
PLSP319	Steroid biosynthesis; cholesterol biosynthesis
PLSP321	Steroid biosynthesis; estrogen biosynthesis
PLSP326	Sulfur metabolism ; glutathione biosynthesis ; glutathione from L-cysteine and L-glutamate
PLSP329	Sulfur metabolism; glutathione metabolism
PLSP332	Terpene metabolism ; lanosterol biosynthesis ; lanosterol from farnesyl-PP
PLSP335	Terpene metabolism; oleoresin biosynthesis
PLSP337	tRNA modification; wybutosine-tRNA(Phe) biosynthesis
