# Supplementary material for: PathLocdb: a comprehensive database for the subcellular localization of metabolic pathways and its application to multiple localization analysis
Source: BMC Genomics. 2010 Dec 2;11(Suppl 4):S13. doi: 10.1186/1471-2164-11-S4-S13 (PMC3005916; doi:10.1186/1471-2164-11-S4-S13)
Supplement: Additional file 3 — The 88 pathways with multiple locations summarized from the multiple locations of their steps are shown in Additional file 3. They provide information on potential existence of transport system between these pathways with different localizations and different steps. [file 1471-2164-11-S4-S13-S3.pdf]

Superpatwhay ID	Organism taxid	Superpathway name	Step	Location	UniProt accession number
PLSP210	10029_rodents	Metabolic intermediate biosynthesis ; mevalonic acid biosynthesis ; (R)-mevalonic acid from acetyl-CoA	step 2/3	cytoplasm	P13704
PLSP210	10029_rodents	Metabolic intermediate biosynthesis ; mevalonic acid biosynthesis ; (R)-mevalonic acid from acetyl-CoA	step 3/3	endoplasmic reticulum	P00347
PLSP14	10090_rodents	Amine and polyamine degradation ; betaine degradation ; sarcosine from betaine	step 1/2	cytoplasm	O35490
PLSP14	10090_rodents	Amine and polyamine degradation ; betaine degradation ; sarcosine from betaine	step 2/2	mitochondria	Q9DBT9
PLSP23	10090_rodents	Amino-acid biosynthesis ; L-cysteine biosynthesis ; L-cysteine from L-homocysteine and L-serine	step 1/2	cytoplasm	Q91WT9
PLSP23	10090_rodents	Amino-acid biosynthesis ; L-cysteine biosynthesis ; L-cysteine from L-homocysteine and L-serine	step 1/2	nucleus	Q91WT9
PLSP23	10090_rodents	Amino-acid biosynthesis ; L-cysteine biosynthesis ; L-cysteine from L-homocysteine and L-serine	step 2/2	cytoplasm	Q8VCN5
PLSP63	10090_rodents	Amino-acid degradation ; L-phenylalanine degradation ; acetoacetic acid and fumarate from L-phenylalanine	step 3/6	cell membrane	P49429
PLSP63	10090_rodents	Amino-acid degradation ; L-phenylalanine degradation ; acetoacetic acid and fumarate from L-phenylalanine	step 3/6	cytoplasm	P49429
PLSP63	10090_rodents	Amino-acid degradation ; L-phenylalanine degradation ; acetoacetic acid and fumarate from L-phenylalanine	step 5/6	cytoplasm	Q9WVL0
PLSP116	10090_rodents	Cofactor biosynthesis ; NAD(+) biosynthesis ; pyridine-2,3-dicarboxylate from L-kynurenine	step 1/3	mitochondria	Q91WN4
PLSP116	10090_rodents	Cofactor biosynthesis ; NAD(+) biosynthesis ; pyridine-2,3-dicarboxylate from L-kynurenine	step 2/3	cytoplasm	Q9CXF0
PLSP116	10090_rodents	Cofactor biosynthesis ; NAD(+) biosynthesis ; pyridine-2,3-dicarboxylate from L-kynurenine	step 3/3	cytoplasm	Q78JT3
PLSP181	10090_rodents	Isoprenoid biosynthesis ; isopentenyl-PP biosynthesis via mevalonic acid pathway ; isopentenyl-PP from (R)-mevalonic acid	step 1/3	cytoplasm	Q9R008
PLSP181	10090_rodents	Isoprenoid biosynthesis ; isopentenyl-PP biosynthesis via mevalonic acid pathway ; isopentenyl-PP from (R)-mevalonic acid	step 1/3	peroxisome	Q9R008
PLSP181	10090_rodents	Isoprenoid biosynthesis ; isopentenyl-PP biosynthesis via mevalonic acid pathway ; isopentenyl-PP from (R)-mevalonic acid	step 2/3	peroxisome	Q9D1G2
PLSP231	10090_rodents	Phospholipid metabolism ; CDP-diacylglycerol biosynthesis ; CDP-diacylglycerol from sn-glycerol 3-phosphate	step 1/3	mitochondria	Q61586
PLSP231	10090_rodents	Phospholipid metabolism ; CDP-diacylglycerol biosynthesis ; CDP-diacylglycerol from sn-glycerol 3-phosphate	step 2/3	endoplasmic reticulum	Q3UN02,Q6NVG1,Q8C0N2
PLSP231	10090_rodents	Phospholipid metabolism ; CDP-diacylglycerol biosynthesis ; CDP-diacylglycerol from sn-glycerol 3-phosphate	step 3/3	cytoplasm	P98191
PLSP231	10090_rodents	Phospholipid metabolism ; CDP-diacylglycerol biosynthesis ; CDP-diacylglycerol from sn-glycerol 3-phosphate	step 3/3	endoplasmic reticulum	P98191

PLSP231	10090_rodents	Phospholipid metabolism ; CDP-diacylglycerol biosynthesis ; CDP-diacylglycerol from sn-glycerol 3-phosphate	step 3/3	mitochondria	Q99L43
PLSP260	10090_rodents	Polyol metabolism ; myo-inositol biosynthesis ; myo-inositol from D-glucose 6-phosphate	step 1/2	cytoplasm	Q9JHU9
PLSP260	10090_rodents	Polyol metabolism ; myo-inositol biosynthesis ; myo-inositol from D-glucose 6-phosphate	step 2/2	cytoplasm	O55023
PLSP260	10090_rodents	Polyol metabolism ; myo-inositol biosynthesis ; myo-inositol from D-glucose 6-phosphate	step 2/2	membrane	Q80V26
PLSP318	10090_rodents	Steroid biosynthesis ; zymosterol biosynthesis ; zymosterol from lanosterol	step 3/6	endoplasmic reticulum	Q9CRA4
PLSP318	10090_rodents	Steroid biosynthesis ; zymosterol biosynthesis ; zymosterol from lanosterol	step 4/6	membrane	Q9R1J0
PLSP318	10090_rodents	Steroid biosynthesis ; zymosterol biosynthesis ; zymosterol from lanosterol	step 5/6	cell membrane	O88736
PLSP332	10090_rodents	Terpene metabolism ; lanosterol biosynthesis ; lanosterol from farnesyl-PP	step 1/3	endoplasmic reticulum	P53798
PLSP332	10090_rodents	Terpene metabolism ; lanosterol biosynthesis ; lanosterol from farnesyl-PP	step 2/3	endoplasmic reticulum	P52019
PLSP332	10090_rodents	Terpene metabolism ; lanosterol biosynthesis ; lanosterol from farnesyl-PP	step 2/3	microsome	P52019
PLSP14	10116_rodents	Amine and polyamine degradation ; betaine degradation ; sarcosine from betaine	step 1/2	cytoplasm	O09171
PLSP14	10116_rodents	Amine and polyamine degradation ; betaine degradation ; sarcosine from betaine	step 2/2	mitochondria	Q63342
PLSP23	10116_rodents	Amino-acid biosynthesis ; L-cysteine biosynthesis ; L-cysteine from L-homocysteine and L-serine	step 1/2	cytoplasm	P32232
PLSP23	10116_rodents	Amino-acid biosynthesis ; L-cysteine biosynthesis ; L-cysteine from L-homocysteine and L-serine	step 1/2	nucleus	P32232
PLSP23	10116_rodents	Amino-acid biosynthesis ; L-cysteine biosynthesis ; L-cysteine from L-homocysteine and L-serine	step 2/2	cytoplasm	P18757
PLSP116	10116_rodents	Cofactor biosynthesis ; NAD(+) biosynthesis ; pyridine-2,3-dicarboxylate from L-kynurenine	step 1/3	mitochondria	O88867
PLSP116	10116_rodents	Cofactor biosynthesis ; NAD(+) biosynthesis ; pyridine-2,3-dicarboxylate from L-kynurenine	step 2/3	cytoplasm	P70712
PLSP116	10116_rodents	Cofactor biosynthesis ; NAD(+) biosynthesis ; pyridine-2,3-dicarboxylate from L-kynurenine	step 3/3	cytoplasm	P46953
PLSP231	10116_rodents	Phospholipid metabolism ; CDP-diacylglycerol biosynthesis ; CDP-diacylglycerol from sn-glycerol 3-phosphate	step 1/3	mitochondria	P97564
PLSP231	10116_rodents	Phospholipid metabolism ; CDP-diacylglycerol biosynthesis ; CDP-diacylglycerol from sn-glycerol 3-phosphate	step 2/3	endoplasmic reticulum	Q4V8J4
PLSP231	10116_rodents	Phospholipid metabolism ; CDP-diacylglycerol biosynthesis ; CDP-diacylglycerol from sn-glycerol 3-phosphate	step 3/3	cytoplasm	O35052

PLSP231	10116_rodents	Phospholipid metabolism ; CDP-diacylglycerol biosynthesis ; CDP-diacylglycerol from sn-glycerol 3-phosphate	step 3/3	endoplasmic reticulum	O35052
PLSP231	10116_rodents	Phospholipid metabolism ; CDP-diacylglycerol biosynthesis ; CDP-diacylglycerol from sn-glycerol 3-phosphate	step 3/3	mitochondria	Q91XU8
PLSP318	10116_rodents	Steroid biosynthesis ; zymosterol biosynthesis ; zymosterol from lanosterol	step 1/6	endoplasmic reticulum	Q64654
PLSP318	10116_rodents	Steroid biosynthesis ; zymosterol biosynthesis ; zymosterol from lanosterol	step 1/6	microsome	Q64654
PLSP318	10116_rodents	Steroid biosynthesis ; zymosterol biosynthesis ; zymosterol from lanosterol	step 3/6	endoplasmic reticulum	O35532
PLSP318	10116_rodents	Steroid biosynthesis ; zymosterol biosynthesis ; zymosterol from lanosterol	step 4/6	membrane	Q5PPL3
PLSP318	10116_rodents	Steroid biosynthesis ; zymosterol biosynthesis ; zymosterol from lanosterol	step 5/6	cell membrane	Q62904
PLSP332	10116_rodents	Terpene metabolism ; lanosterol biosynthesis ; lanosterol from farnesyl-PP	step 1/3	endoplasmic reticulum	Q02769
PLSP332	10116_rodents	Terpene metabolism ; lanosterol biosynthesis ; lanosterol from farnesyl-PP	step 2/3	endoplasmic reticulum	P52020
PLSP332	10116_rodents	Terpene metabolism ; lanosterol biosynthesis ; lanosterol from farnesyl-PP	step 2/3	microsome	P52020
PLSP116	13684_fungi	Cofactor biosynthesis ; NAD(+) biosynthesis ; pyridine-2,3-dicarboxylate from L-kynurenine	step 1/3	mitochondria	Q0V5K1
PLSP116	13684_fungi	Cofactor biosynthesis ; NAD(+) biosynthesis ; pyridine-2,3-dicarboxylate from L-kynurenine	step 2/3	cytoplasm	Q0UIN2,Q0UZK0
PLSP116	162425_fungi	Cofactor biosynthesis ; NAD(+) biosynthesis ; pyridine-2,3-dicarboxylate from L-kynurenine	step 1/3	mitochondria	Q5B2N0
PLSP116	162425_fungi	Cofactor biosynthesis ; NAD(+) biosynthesis ; pyridine-2,3-dicarboxylate from L-kynurenine	step 2/3	cytoplasm	Q5B0H8,Q5BC73
PLSP116	331117_fungi	Cofactor biosynthesis ; NAD(+) biosynthesis ; pyridine-2,3-dicarboxylate from L-kynurenine	step 1/3	mitochondria	A1DMD5
PLSP116	331117_fungi	Cofactor biosynthesis ; NAD(+) biosynthesis ; pyridine-2,3-dicarboxylate from L-kynurenine	step 2/3	cytoplasm	A1DGW4,A1CWY1
PLSP116	331117_fungi	Cofactor biosynthesis ; NAD(+) biosynthesis ; pyridine-2,3-dicarboxylate from L-kynurenine	step 3/3	cytoplasm	A1DB76
PLSP116	33169_fungi	Cofactor biosynthesis ; NAD(+) biosynthesis ; pyridine-2,3-dicarboxylate from L-kynurenine	step 1/3	mitochondria	Q751I2
PLSP116	33169_fungi	Cofactor biosynthesis ; NAD(+) biosynthesis ; pyridine-2,3-dicarboxylate from L-kynurenine	step 2/3	cytoplasm	Q750P5
PLSP116	33169_fungi	Cofactor biosynthesis ; NAD(+) biosynthesis ; pyridine-2,3-dicarboxylate from L-kynurenine	step 3/3	cytoplasm	Q75CP6
PLSP332	33169_fungi	Terpene metabolism ; lanosterol biosynthesis ; lanosterol from farnesyl-PP	step 1/3	endoplasmic reticulum	Q752X9
PLSP332	33169_fungi	Terpene metabolism ; lanosterol biosynthesis ; lanosterol from farnesyl-PP	step 2/3	endoplasmic reticulum	Q75F69

PLSP332	33169_fungi	Terpene metabolism ; lanosterol biosynthesis ; lanosterol from farnesyl-PP	step 2/3	microsome	Q75F69
PLSP116	341663_fungi	Cofactor biosynthesis ; NAD(+) biosynthesis ; pyridine-2,3-dicarboxylate from L-kynurenine	step 1/3	mitochondria	Q0CRI5
PLSP116	341663_fungi	Cofactor biosynthesis ; NAD(+) biosynthesis ; pyridine-2,3-dicarboxylate from L-kynurenine	step 2/3	cytoplasm	Q0CPB0,Q0CZX6
PLSP116	341663_fungi	Cofactor biosynthesis ; NAD(+) biosynthesis ; pyridine-2,3-dicarboxylate from L-kynurenine	step 3/3	cytoplasm	Q0D1U6
PLSP62	3702_plants	Amino-acid degradation ; L-lysine degradation via saccharopine pathway ; glutaryl-CoA from L-lysine	step 1/6	cytoplasm	Q9SMZ4
PLSP62	3702_plants	Amino-acid degradation ; L-lysine degradation via saccharopine pathway ; glutaryl-CoA from L-lysine	step 2/6	cytoplasm	Q9SMZ4
PLSP62	3702_plants	Amino-acid degradation ; L-lysine degradation via saccharopine pathway ; glutaryl-CoA from L-lysine	step 2/6	mitochondria	Q8LGI2
PLSP210	3702_plants	Metabolic intermediate biosynthesis ; mevalonic acid biosynthesis ; (R)-mevalonic acid from acetyl-CoA	step 1/3	cytoplasm	Q8S4Y1
PLSP210	3702_plants	Metabolic intermediate biosynthesis ; mevalonic acid biosynthesis ; (R)-mevalonic acid from acetyl-CoA	step 3/3	endoplasmic reticulum	P14891,P43256
PLSP301	3702_plants	Purine metabolism ; uric acid degradation ; (S)-allantoin from uric acid	step 1/3	peroxisome	O04420
PLSP301	3702_plants	Purine metabolism ; uric acid degradation ; (S)-allantoin from uric acid	step 2/3	cell membrane	Q9LVM5
PLSP301	3702_plants	Purine metabolism ; uric acid degradation ; (S)-allantoin from uric acid	step 3/3	cell membrane	Q9LVM5
PLSP332	3702_plants	Terpene metabolism ; lanosterol biosynthesis ; lanosterol from farnesyl-PP	step 1/3	endoplasmic reticulum	P53799
PLSP332	3702_plants	Terpene metabolism ; lanosterol biosynthesis ; lanosterol from farnesyl-PP	step 2/3	membrane	O65404,O65402,O65403
PLSP116	38033_fungi	Cofactor biosynthesis ; NAD(+) biosynthesis ; pyridine-2,3-dicarboxylate from L-kynurenine	step 1/3	mitochondria	Q2GQG8
PLSP116	38033_fungi	Cofactor biosynthesis ; NAD(+) biosynthesis ; pyridine-2,3-dicarboxylate from L-kynurenine	step 2/3	cytoplasm	Q2H9P7,Q2H7G2
PLSP116	38033_fungi	Cofactor biosynthesis ; NAD(+) biosynthesis ; pyridine-2,3-dicarboxylate from L-kynurenine	step 3/3	cytoplasm	Q2HD63
PLSP6	4058_plants	Alkaloid biosynthesis ; vindoline biosynthesis ; vindoline from tabersonine	step 1/6	endoplasmic reticulum	P98183
PLSP6	4058_plants	Alkaloid biosynthesis ; vindoline biosynthesis ; vindoline from tabersonine	step 5/6	cytoplasm	O04847
PLSP159	4081_plants	Glycan metabolism ; pectin degradation ; 2-dehydro-3-deoxy-D-gluconic acid from pectin	step 1/5	cell wall	P14280,P09607,Q96575,Q96576,Q43143
PLSP159	4081_plants	Glycan metabolism ; pectin degradation ; 2-dehydro-3-deoxy-D-gluconic acid from pectin	step 1/5	extracellular	P14280,P09607,Q96575,Q96576,Q43143
PLSP159	4081_plants	Glycan metabolism ; pectin degradation ; 2-dehydro-3-deoxy-D-gluconic acid from pectin	step 2/5	extracellular	P24396
PLSP116	425011_fungi	Cofactor biosynthesis ; NAD(+) biosynthesis ; pyridine-2,3-dicarboxylate from L-kynurenine	step 1/3	mitochondria	A2Q9N7,A2QMH1,A2QPD9

PLSP116	425011_fungi	Cofactor biosynthesis ; NAD(+) biosynthesis ; pyridine-2,3-dicarboxylate from L-kynurenine	step 2/3	cytoplasm	A2R7T0,A2QJI5
PLSP116	425011_fungi	Cofactor biosynthesis ; NAD(+) biosynthesis ; pyridine-2,3-dicarboxylate from L-kynurenine	step 3/3	cytoplasm	A2R8S7
PLSP116	44689_invertebrates	Cofactor biosynthesis ; NAD(+) biosynthesis ; pyridine-2,3-dicarboxylate from L-kynurenine	step 1/3	mitochondria	Q54RE8
PLSP116	44689_invertebrates	Cofactor biosynthesis ; NAD(+) biosynthesis ; pyridine-2,3-dicarboxylate from L-kynurenine	step 2/3	cytoplasm	Q54Q04
PLSP116	44689_invertebrates	Cofactor biosynthesis ; NAD(+) biosynthesis ; pyridine-2,3-dicarboxylate from L-kynurenine	step 3/3	cytoplasm	Q54S02
PLSP116	451804_fungi	Cofactor biosynthesis ; NAD(+) biosynthesis ; pyridine-2,3-dicarboxylate from L-kynurenine	step 1/3	mitochondria	B0Y7C3
PLSP116	451804_fungi	Cofactor biosynthesis ; NAD(+) biosynthesis ; pyridine-2,3-dicarboxylate from L-kynurenine	step 2/3	cytoplasm	B0XS72,B0Y6H2
PLSP116	451804_fungi	Cofactor biosynthesis ; NAD(+) biosynthesis ; pyridine-2,3-dicarboxylate from L-kynurenine	step 3/3	cytoplasm	B0Y9Z9,B0XVP0
PLSP29	4896_fungi	Amino-acid biosynthesis ; L-isoleucine biosynthesis ; L-isoleucine from 2-oxobutanoate	step 1/4	cytoplasm	O60086
PLSP29	4896_fungi	Amino-acid biosynthesis ; L-isoleucine biosynthesis ; L-isoleucine from 2-oxobutanoate	step 1/4	mitochondria	P36620
PLSP29	4896_fungi	Amino-acid biosynthesis ; L-isoleucine biosynthesis ; L-isoleucine from 2-oxobutanoate	step 2/4	mitochondria	P78827
PLSP29	4896_fungi	Amino-acid biosynthesis ; L-isoleucine biosynthesis ; L-isoleucine from 2-oxobutanoate	step 3/4	mitochondria	Q10318
PLSP31	4896_fungi	Amino-acid biosynthesis ; L-lysine biosynthesis via AAA pathway ; L-alpha-aminoadipate from 2-oxoglutarate	step 1/4	mitochondria	Q9Y823
PLSP31	4896_fungi	Amino-acid biosynthesis ; L-lysine biosynthesis via AAA pathway ; L-alpha-aminoadipate from 2-oxoglutarate	step 2/4	mitochondria	Q9UT74
PLSP31	4896_fungi	Amino-acid biosynthesis ; L-lysine biosynthesis via AAA pathway ; L-alpha-aminoadipate from 2-oxoglutarate	step 3/4	cytoplasm	O14104
PLSP51	4896_fungi	Amino-acid biosynthesis ; L-valine biosynthesis ; L-valine from pyruvate	step 1/4	cytoplasm	O60086
PLSP51	4896_fungi	Amino-acid biosynthesis ; L-valine biosynthesis ; L-valine from pyruvate	step 1/4	mitochondria	P36620
PLSP51	4896_fungi	Amino-acid biosynthesis ; L-valine biosynthesis ; L-valine from pyruvate	step 2/4	mitochondria	P78827
PLSP51	4896_fungi	Amino-acid biosynthesis ; L-valine biosynthesis ; L-valine from pyruvate	step 3/4	mitochondria	Q10318
PLSP85	4896_fungi	Carbohydrate degradation ; glycolysis ; pyruvate from D-glyceraldehyde 3-phosphate	step 1/5	cytoplasm	P78958,O43026
PLSP85	4896_fungi	Carbohydrate degradation ; glycolysis ; pyruvate from D-glyceraldehyde 3-phosphate	step 2/5	cytoplasm	O60101
PLSP85	4896_fungi	Carbohydrate degradation ; glycolysis ; pyruvate from D-glyceraldehyde 3-phosphate	step 3/5	cytoplasm	O94461,Q9UT63
PLSP85	4896_fungi	Carbohydrate degradation ; glycolysis ; pyruvate from D-glyceraldehyde 3-phosphate	step 3/5	nucleus	O94461,Q9UT63,O94420

PLSP85	4896_fungi	Carbohydrate degradation ; glycolysis ; pyruvate from D-glyceraldehyde 3-phosphate	step 4/5	cytoplasm	P40370,Q8NKC2
PLSP209	4896_fungi	Metabolic intermediate biosynthesis ; chorismate biosynthesis ; chorismate from D-erythrose 4-phosphate and PEP	step 1/7	cytoplasm	Q9UT09
PLSP209	4896_fungi	Metabolic intermediate biosynthesis ; chorismate biosynthesis ; chorismate from D-erythrose 4-phosphate and PEP	step 1/7	nucleus	Q9UT09
PLSP209	4896_fungi	Metabolic intermediate biosynthesis ; chorismate biosynthesis ; chorismate from D-erythrose 4-phosphate and PEP	step 2/7	cytoplasm	Q9P7R0
PLSP209	4896_fungi	Metabolic intermediate biosynthesis ; chorismate biosynthesis ; chorismate from D-erythrose 4-phosphate and PEP	step 3/7	cytoplasm	Q9P7R0
PLSP209	4896_fungi	Metabolic intermediate biosynthesis ; chorismate biosynthesis ; chorismate from D-erythrose 4-phosphate and PEP	step 4/7	cytoplasm	Q9P7R0
PLSP209	4896_fungi	Metabolic intermediate biosynthesis ; chorismate biosynthesis ; chorismate from D-erythrose 4-phosphate and PEP	step 5/7	cytoplasm	Q9P7R0
PLSP209	4896_fungi	Metabolic intermediate biosynthesis ; chorismate biosynthesis ; chorismate from D-erythrose 4-phosphate and PEP	step 6/7	cytoplasm	Q9P7R0
PLSP210	4896_fungi	Metabolic intermediate biosynthesis ; mevalonic acid biosynthesis ; (R)-mevalonic acid from acetyl-CoA	step 1/3	cytoplasm	Q9UQW6
PLSP210	4896_fungi	Metabolic intermediate biosynthesis ; mevalonic acid biosynthesis ; (R)-mevalonic acid from acetyl-CoA	step 3/3	endoplasmic reticulum	Q10283
PLSP318	4896_fungi	Steroid biosynthesis ; zymosterol biosynthesis ; zymosterol from lanosterol	step 1/6	endoplasmic reticulum	Q09736
PLSP318	4896_fungi	Steroid biosynthesis ; zymosterol biosynthesis ; zymosterol from lanosterol	step 2/6	membrane	Q09195
PLSP318	4896_fungi	Steroid biosynthesis ; zymosterol biosynthesis ; zymosterol from lanosterol	step 3/6	endoplasmic reticulum	Q9UUH4
PLSP318	4896_fungi	Steroid biosynthesis ; zymosterol biosynthesis ; zymosterol from lanosterol	step 4/6	endoplasmic reticulum	O43050
PLSP324	4896_fungi	Steroid metabolism ; ergosterol biosynthesis ; ergosterol from zymosterol	step 2/5	endoplasmic reticulum	P87113
PLSP324	4896_fungi	Steroid metabolism ; ergosterol biosynthesis ; ergosterol from zymosterol	step 3/5	endoplasmic reticulum	O94457,O13666
PLSP324	4896_fungi	Steroid metabolism ; ergosterol biosynthesis ; ergosterol from zymosterol	step 5/5	membrane	P36209
PLSP332	4896_fungi	Terpene metabolism ; lanosterol biosynthesis ; lanosterol from farnesyl-PP	step 1/3	endoplasmic reticulum	P36596
PLSP332	4896_fungi	Terpene metabolism ; lanosterol biosynthesis ; lanosterol from farnesyl-PP	step 2/3	endoplasmic reticulum	Q9C1W3
PLSP332	4896_fungi	Terpene metabolism ; lanosterol biosynthesis ; lanosterol from farnesyl-PP	step 2/3	microsome	Q9C1W3
PLSP116	4924_fungi	Cofactor biosynthesis ; NAD(+) biosynthesis ; pyridine-2,3-dicarboxylate from L-kynurenine	step 1/3	mitochondria	A3LNF8
PLSP116	4924_fungi	Cofactor biosynthesis ; NAD(+) biosynthesis ; pyridine-2,3-dicarboxylate from L-kynurenine	step 2/3	cytoplasm	A3LQD7
PLSP116	4924_fungi	Cofactor biosynthesis ; NAD(+) biosynthesis ; pyridine-2,3-dicarboxylate from L-kynurenine	step 3/3	cytoplasm	A3LP72
PLSP30	4932_fungi	Amino-acid biosynthesis ; L-leucine biosynthesis ; L-leucine from 3-methyl-2-oxobutanoate	step 1/4	cytoplasm	P06208

PLSP30	4932_fungi	Amino-acid biosynthesis ; L-leucine biosynthesis ; L-leucine from 3-methyl-2-oxobutanoate	step 1/4	mitochondria	P06208,Q12166
PLSP30	4932_fungi	Amino-acid biosynthesis ; L-leucine biosynthesis ; L-leucine from 3-methyl-2-oxobutanoate	step 3/4	cytoplasm	P04173
PLSP31	4932_fungi	Amino-acid biosynthesis ; L-lysine biosynthesis via AAA pathway ; L-alpha-aminoadipate from 2-oxoglutarate	step 1/4	cytoplasm	P48570
PLSP31	4932_fungi	Amino-acid biosynthesis ; L-lysine biosynthesis via AAA pathway ; L-alpha-aminoadipate from 2-oxoglutarate	step 1/4	mitochondria	Q12122
PLSP31	4932_fungi	Amino-acid biosynthesis ; L-lysine biosynthesis via AAA pathway ; L-alpha-aminoadipate from 2-oxoglutarate	step 2/4	mitochondria	P49367
PLSP31	4932_fungi	Amino-acid biosynthesis ; L-lysine biosynthesis via AAA pathway ; L-alpha-aminoadipate from 2-oxoglutarate	step 3/4	mitochondria	P40495
PLSP85	4932_fungi	Carbohydrate degradation ; glycolysis ; pyruvate from D-glyceraldehyde 3-phosphate	step 1/5	cytoplasm	P00360,P00358,P00359
PLSP85	4932_fungi	Carbohydrate degradation ; glycolysis ; pyruvate from D-glyceraldehyde 3-phosphate	step 2/5	cytoplasm	P00560
PLSP85	4932_fungi	Carbohydrate degradation ; glycolysis ; pyruvate from D-glyceraldehyde 3-phosphate	step 3/5	cytoplasm	Q12040,Q12008
PLSP85	4932_fungi	Carbohydrate degradation ; glycolysis ; pyruvate from D-glyceraldehyde 3-phosphate	step 3/5	nucleus	Q12040
PLSP85	4932_fungi	Carbohydrate degradation ; glycolysis ; pyruvate from D-glyceraldehyde 3-phosphate	step 4/5	cytoplasm	P00924,P00925
PLSP210	4932_fungi	Metabolic intermediate biosynthesis ; mevalonic acid biosynthesis ; (R)-mevalonic acid from acetyl-CoA	step 1/3	cytoplasm	P41338
PLSP210	4932_fungi	Metabolic intermediate biosynthesis ; mevalonic acid biosynthesis ; (R)-mevalonic acid from acetyl-CoA	step 3/3	endoplasmic reticulum	P12683,P12684
PLSP260	4932_fungi	Polyol metabolism ; myo-inositol biosynthesis ; myo-inositol from D-glucose 6-phosphate	step 1/2	cytoplasm	P11986
PLSP260	4932_fungi	Polyol metabolism ; myo-inositol biosynthesis ; myo-inositol from D-glucose 6-phosphate	step 2/2	cytoplasm	P38710
PLSP260	4932_fungi	Polyol metabolism ; myo-inositol biosynthesis ; myo-inositol from D-glucose 6-phosphate	step 2/2	nucleus	P38710
PLSP318	4932_fungi	Steroid biosynthesis ; zymosterol biosynthesis ; zymosterol from lanosterol	step 1/6	membrane	P10614
PLSP318	4932_fungi	Steroid biosynthesis ; zymosterol biosynthesis ; zymosterol from lanosterol	step 2/6	membrane	P32462
PLSP318	4932_fungi	Steroid biosynthesis ; zymosterol biosynthesis ; zymosterol from lanosterol	step 3/6	endoplasmic reticulum	P53045
PLSP318	4932_fungi	Steroid biosynthesis ; zymosterol biosynthesis ; zymosterol from lanosterol	step 4/6	endoplasmic reticulum	P53199
PLSP318	4932_fungi	Steroid biosynthesis ; zymosterol biosynthesis ; zymosterol from lanosterol	step 5/6	endoplasmic reticulum	Q12452
PLSP324	4932_fungi	Steroid metabolism ; ergosterol biosynthesis ; ergosterol from zymosterol	step 2/5	endoplasmic reticulum	P32352
PLSP324	4932_fungi	Steroid metabolism ; ergosterol biosynthesis ; ergosterol from zymosterol	step 3/5	endoplasmic reticulum	P32353
PLSP324	4932_fungi	Steroid metabolism ; ergosterol biosynthesis ; ergosterol from zymosterol	step 5/5	membrane	P25340

PLSP332	4932_fungi	Terpene metabolism ; lanosterol biosynthesis ; lanosterol from farnesyl-PP	step 1/3	endoplasmic reticulum	P29704
PLSP332	4932_fungi	Terpene metabolism ; lanosterol biosynthesis ; lanosterol from farnesyl-PP	step 2/3	endoplasmic reticulum	P32476
PLSP332	4932_fungi	Terpene metabolism ; lanosterol biosynthesis ; lanosterol from farnesyl-PP	step 2/3	microsome	P32476
PLSP332	4932_fungi	Terpene metabolism ; lanosterol biosynthesis ; lanosterol from farnesyl-PP	step 3/3	endoplasmic reticulum	P38604
PLSP116	4952_fungi	Cofactor biosynthesis ; NAD(+) biosynthesis ; pyridine-2,3-dicarboxylate from L-kynurenine	step 1/3	mitochondria	Q6C9M8
PLSP116	4952_fungi	Cofactor biosynthesis ; NAD(+) biosynthesis ; pyridine-2,3-dicarboxylate from L-kynurenine	step 2/3	cytoplasm	Q6CDM0
PLSP116	4952_fungi	Cofactor biosynthesis ; NAD(+) biosynthesis ; pyridine-2,3-dicarboxylate from L-kynurenine	step 3/3	cytoplasm	Q6CFX1
PLSP116	4959_fungi	Cofactor biosynthesis ; NAD(+) biosynthesis ; pyridine-2,3-dicarboxylate from L-kynurenine	step 1/3	mitochondria	Q6BV21
PLSP116	4959_fungi	Cofactor biosynthesis ; NAD(+) biosynthesis ; pyridine-2,3-dicarboxylate from L-kynurenine	step 2/3	cytoplasm	Q6BI19
PLSP116	5057_fungi	Cofactor biosynthesis ; NAD(+) biosynthesis ; pyridine-2,3-dicarboxylate from L-kynurenine	step 1/3	mitochondria	A1CT23
PLSP116	5057_fungi	Cofactor biosynthesis ; NAD(+) biosynthesis ; pyridine-2,3-dicarboxylate from L-kynurenine	step 2/3	cytoplasm	A1C688,A1CHT0
PLSP116	5057_fungi	Cofactor biosynthesis ; NAD(+) biosynthesis ; pyridine-2,3-dicarboxylate from L-kynurenine	step 3/3	cytoplasm	A1C408,A1C874
PLSP116	5062_fungi	Cofactor biosynthesis ; NAD(+) biosynthesis ; pyridine-2,3-dicarboxylate from L-kynurenine	step 1/3	mitochondria	Q2UPP1
PLSP116	5062_fungi	Cofactor biosynthesis ; NAD(+) biosynthesis ; pyridine-2,3-dicarboxylate from L-kynurenine	step 2/3	cytoplasm	Q2U038,Q2UJE8
PLSP116	5062_fungi	Cofactor biosynthesis ; NAD(+) biosynthesis ; pyridine-2,3-dicarboxylate from L-kynurenine	step 3/3	cytoplasm	Q2UHT9,Q2UB88
PLSP116	5085_fungi	Cofactor biosynthesis ; NAD(+) biosynthesis ; pyridine-2,3-dicarboxylate from L-kynurenine	step 1/3	mitochondria	Q4WN75
PLSP116	5085_fungi	Cofactor biosynthesis ; NAD(+) biosynthesis ; pyridine-2,3-dicarboxylate from L-kynurenine	step 2/3	cytoplasm	Q4X1D4,Q4WPN0
PLSP116	5085_fungi	Cofactor biosynthesis ; NAD(+) biosynthesis ; pyridine-2,3-dicarboxylate from L-kynurenine	step 3/3	cytoplasm	Q4WCF1,Q4WZC3
PLSP116	5141_fungi	Cofactor biosynthesis ; NAD(+) biosynthesis ; pyridine-2,3-dicarboxylate from L-kynurenine	step 1/3	mitochondria	Q7S3C9
PLSP116	5141_fungi	Cofactor biosynthesis ; NAD(+) biosynthesis ; pyridine-2,3-dicarboxylate from L-kynurenine	step 2/3	cytoplasm	Q7RXY2,Q7S332
PLSP116	5141_fungi	Cofactor biosynthesis ; NAD(+) biosynthesis ; pyridine-2,3-dicarboxylate from L-kynurenine	step 3/3	cytoplasm	Q7SDX3

PLSP116	5207_fungi	Cofactor biosynthesis ; NAD(+) biosynthesis ; pyridine-2,3-dicarboxylate from L-kynurenine	step 1/3	mitochondria	Q55W30
PLSP116	5207_fungi	Cofactor biosynthesis ; NAD(+) biosynthesis ; pyridine-2,3-dicarboxylate from L-kynurenine	step 2/3	cytoplasm	Q5KK77
PLSP116	5207_fungi	Cofactor biosynthesis ; NAD(+) biosynthesis ; pyridine-2,3-dicarboxylate from L-kynurenine	step 3/3	cytoplasm	Q5KLD9
PLSP116	5270_fungi	Cofactor biosynthesis ; NAD(+) biosynthesis ; pyridine-2,3-dicarboxylate from L-kynurenine	step 1/3	mitochondria	Q4P0N0
PLSP116	5270_fungi	Cofactor biosynthesis ; NAD(+) biosynthesis ; pyridine-2,3-dicarboxylate from L-kynurenine	step 3/3	cytoplasm	Q4P2Q7
PLSP116	5476_fungi	Cofactor biosynthesis ; NAD(+) biosynthesis ; pyridine-2,3-dicarboxylate from L-kynurenine	step 1/3	mitochondria	Q5A7M3
PLSP116	5476_fungi	Cofactor biosynthesis ; NAD(+) biosynthesis ; pyridine-2,3-dicarboxylate from L-kynurenine	step 2/3	cytoplasm	Q59QC4
PLSP116	5476_fungi	Cofactor biosynthesis ; NAD(+) biosynthesis ; pyridine-2,3-dicarboxylate from L-kynurenine	step 3/3	cytoplasm	Q59K86
PLSP318	5476_fungi	Steroid biosynthesis ; zymosterol biosynthesis ; zymosterol from lanosterol	step 1/6	membrane	P10613
PLSP318	5476_fungi	Steroid biosynthesis ; zymosterol biosynthesis ; zymosterol from lanosterol	step 3/6	endoplasmic reticulum	O59933
PLSP332	5476_fungi	Terpene metabolism ; lanosterol biosynthesis ; lanosterol from farnesyl-PP	step 1/3	endoplasmic reticulum	P78589
PLSP332	5476_fungi	Terpene metabolism ; lanosterol biosynthesis ; lanosterol from farnesyl-PP	step 2/3	endoplasmic reticulum	Q92206
PLSP332	5476_fungi	Terpene metabolism ; lanosterol biosynthesis ; lanosterol from farnesyl-PP	step 2/3	microsome	Q92206
PLSP332	5478_fungi	Terpene metabolism ; lanosterol biosynthesis ; lanosterol from farnesyl-PP	step 1/3	endoplasmic reticulum	Q9HGZ6
PLSP332	5478_fungi	Terpene metabolism ; lanosterol biosynthesis ; lanosterol from farnesyl-PP	step 2/3	endoplasmic reticulum	O13306
PLSP332	5478_fungi	Terpene metabolism ; lanosterol biosynthesis ; lanosterol from farnesyl-PP	step 2/3	microsome	O13306
PLSP116	6238_invertebrates	Cofactor biosynthesis ; NAD(+) biosynthesis ; pyridine-2,3-dicarboxylate from L-kynurenine	step 1/3	mitochondria	A8Y432
PLSP116	6238_invertebrates	Cofactor biosynthesis ; NAD(+) biosynthesis ; pyridine-2,3-dicarboxylate from L-kynurenine	step 2/3	cytoplasm	A8XKT0
PLSP116	6238_invertebrates	Cofactor biosynthesis ; NAD(+) biosynthesis ; pyridine-2,3-dicarboxylate from L-kynurenine	step 3/3	cytoplasm	Q60W34
PLSP116	6239_invertebrates	Cofactor biosynthesis ; NAD(+) biosynthesis ; pyridine-2,3-dicarboxylate from L-kynurenine	step 1/3	mitochondria	Q21795
PLSP116	6239_invertebrates	Cofactor biosynthesis ; NAD(+) biosynthesis ; pyridine-2,3-dicarboxylate from L-kynurenine	step 2/3	cytoplasm	Q18026
PLSP116	6239_invertebrates	Cofactor biosynthesis ; NAD(+) biosynthesis ; pyridine-2,3-dicarboxylate from L-kynurenine	step 3/3	cytoplasm	Q19341

PLSP231	6239_invertebrates	Phospholipid metabolism ; CDP-diacylglycerol biosynthesis ; CDP-diacylglycerol from sn-glycerol 3-phosphate	step 1/3	mitochondria	Q22949
PLSP231	6239_invertebrates	Phospholipid metabolism ; CDP-diacylglycerol biosynthesis ; CDP-diacylglycerol from sn-glycerol 3-phosphate	step 2/3	membrane	Q11087,Q93841,Q22267
PLSP231	6239_invertebrates	Phospholipid metabolism ; CDP-diacylglycerol biosynthesis ; CDP-diacylglycerol from sn-glycerol 3-phosphate	step 3/3	membrane	P53439
PLSP260	7227_invertebrates	Polyol metabolism ; myo-inositol biosynthesis ; myo-inositol from D-glucose 6-phosphate	step 1/2	cytoplasm	O97477
PLSP260	7227_invertebrates	Polyol metabolism ; myo-inositol biosynthesis ; myo-inositol from D-glucose 6-phosphate	step 2/2	membrane	Q9VYF2
PLSP303	7227_invertebrates	Pyrimidine metabolism ; UMP biosynthesis via de novo pathway ; UMP from HCO(3)(-)	step 1/6	cytoplasm	P05990
PLSP303	7227_invertebrates	Pyrimidine metabolism ; UMP biosynthesis via de novo pathway ; UMP from HCO(3)(-)	step 2/6	cytoplasm	P05990
PLSP303	7227_invertebrates	Pyrimidine metabolism ; UMP biosynthesis via de novo pathway ; UMP from HCO(3)(-)	step 3/6	cytoplasm	P05990
PLSP303	7227_invertebrates	Pyrimidine metabolism ; UMP biosynthesis via de novo pathway ; UMP from HCO(3)(-)	step 4/6	mitochondria	P32748
PLSP116	7955_vertebrates	Cofactor biosynthesis ; NAD(+) biosynthesis ; pyridine-2,3-dicarboxylate from L-kynurenine	step 1/3	mitochondria	Q1RLY6
PLSP116	7955_vertebrates	Cofactor biosynthesis ; NAD(+) biosynthesis ; pyridine-2,3-dicarboxylate from L-kynurenine	step 3/3	cytoplasm	Q5U3F8
PLSP260	8355_vertebrates	Polyol metabolism ; myo-inositol biosynthesis ; myo-inositol from D-glucose 6-phosphate	step 1/2	cytoplasm	Q7ZXY0,Q6DDT1
PLSP260	8355_vertebrates	Polyol metabolism ; myo-inositol biosynthesis ; myo-inositol from D-glucose 6-phosphate	step 2/2	cytoplasm	P29219
PLSP260	8355_vertebrates	Polyol metabolism ; myo-inositol biosynthesis ; myo-inositol from D-glucose 6-phosphate	step 2/2	membrane	Q6NTW5
PLSP116	8364_vertebrates	Cofactor biosynthesis ; NAD(+) biosynthesis ; pyridine-2,3-dicarboxylate from L-kynurenine	step 1/3	mitochondria	Q6DIZ8
PLSP116	8364_vertebrates	Cofactor biosynthesis ; NAD(+) biosynthesis ; pyridine-2,3-dicarboxylate from L-kynurenine	step 3/3	cytoplasm	Q6DIZ0
PLSP23	9541_mammals	Amino-acid biosynthesis ; L-cysteine biosynthesis ; L-cysteine from L-homocysteine and L-serine	step 1/2	cytoplasm	Q58H57
PLSP23	9541_mammals	Amino-acid biosynthesis ; L-cysteine biosynthesis ; L-cysteine from L-homocysteine and L-serine	step 1/2	nucleus	Q58H57
PLSP23	9541_mammals	Amino-acid biosynthesis ; L-cysteine biosynthesis ; L-cysteine from L-homocysteine and L-serine	step 2/2	cytoplasm	Q60HG7
PLSP318	9601_mammals	Steroid biosynthesis ; zymosterol biosynthesis ; zymosterol from lanosterol	step 1/6	endoplasmic reticulum	Q5RE72
PLSP318	9601_mammals	Steroid biosynthesis ; zymosterol biosynthesis ; zymosterol from lanosterol	step 1/6	microsome	Q5RE72
PLSP318	9601_mammals	Steroid biosynthesis ; zymosterol biosynthesis ; zymosterol from lanosterol	step 3/6	endoplasmic reticulum	Q5R574
PLSP14	9606_human	Amine and polyamine degradation ; betaine degradation ; sarcosine from betaine	step 1/2	cytoplasm	Q93088

PLSP14	9606_human	Amine and polyamine degradation ; betaine degradation ; sarcosine from betaine	step 2/2	mitochondria	Q9UI17
PLSP23	9606_human	Amino-acid biosynthesis ; L-cysteine biosynthesis ; L-cysteine from L-homocysteine and L-serine	step 1/2	cytoplasm	P35520
PLSP23	9606_human	Amino-acid biosynthesis ; L-cysteine biosynthesis ; L-cysteine from L-homocysteine and L-serine	step 1/2	nucleus	P35520
PLSP23	9606_human	Amino-acid biosynthesis ; L-cysteine biosynthesis ; L-cysteine from L-homocysteine and L-serine	step 2/2	cytoplasm	P32929
PLSP116	9606_human	Cofactor biosynthesis ; NAD(+) biosynthesis ; pyridine-2,3-dicarboxylate from L-kynurenine	step 1/3	mitochondria	O15229
PLSP116	9606_human	Cofactor biosynthesis ; NAD(+) biosynthesis ; pyridine-2,3-dicarboxylate from L-kynurenine	step 2/3	cytoplasm	Q16719
PLSP116	9606_human	Cofactor biosynthesis ; NAD(+) biosynthesis ; pyridine-2,3-dicarboxylate from L-kynurenine	step 3/3	cytoplasm	P46952
PLSP121	9606_human	Cofactor biosynthesis ; coenzyme A biosynthesis ; coenzyme A from pantothenate	step 1/5	cytoplasm	Q9BZ23,Q8TEO4,Q9H999,Q9NVE7
PLSP121	9606_human	Cofactor biosynthesis ; coenzyme A biosynthesis ; coenzyme A from pantothenate	step 1/5	mitochondria	Q9BZ23
PLSP121	9606_human	Cofactor biosynthesis ; coenzyme A biosynthesis ; coenzyme A from pantothenate	step 4/5	cytoplasm	Q13057
PLSP121	9606_human	Cofactor biosynthesis ; coenzyme A biosynthesis ; coenzyme A from pantothenate	step 5/5	cytoplasm	Q13057
PLSP181	9606_human	Isoprenoid biosynthesis ; isopentenyl-PP biosynthesis via mevalonic acid pathway ; isopentenyl-PP from (R)-mevalonic acid	step 1/3	cytoplasm	Q03426
PLSP181	9606_human	Isoprenoid biosynthesis ; isopentenyl-PP biosynthesis via mevalonic acid pathway ; isopentenyl-PP from (R)-mevalonic acid	step 1/3	peroxisome	Q03426
PLSP181	9606_human	Isoprenoid biosynthesis ; isopentenyl-PP biosynthesis via mevalonic acid pathway ; isopentenyl-PP from (R)-mevalonic acid	step 2/3	peroxisome	Q15126
PLSP231	9606_human	Phospholipid metabolism ; CDP-diacylglycerol biosynthesis ; CDP-diacylglycerol from sn-glycerol 3-phosphate	step 1/3	mitochondria	Q9HCL2
PLSP231	9606_human	Phospholipid metabolism ; CDP-diacylglycerol biosynthesis ; CDP-diacylglycerol from sn-glycerol 3-phosphate	step 2/3	endoplasmic reticulum	Q6UWP7,Q643R3,Q53EU6
PLSP231	9606_human	Phospholipid metabolism ; CDP-diacylglycerol biosynthesis ; CDP-diacylglycerol from sn-glycerol 3-phosphate	step 3/3	cytoplasm	Q92903
PLSP231	9606_human	Phospholipid metabolism ; CDP-diacylglycerol biosynthesis ; CDP-diacylglycerol from sn-glycerol 3-phosphate	step 3/3	endoplasmic reticulum	Q92903
PLSP231	9606_human	Phospholipid metabolism ; CDP-diacylglycerol biosynthesis ; CDP-diacylglycerol from sn-glycerol 3-phosphate	step 3/3	mitochondria	O95674
PLSP260	9606_human	Polyol metabolism ; myo-inositol biosynthesis ; myo-inositol from D-glucose 6-phosphate	step 1/2	cytoplasm	Q9NPH2

PLSP260	9606_human	Polyol metabolism ; myo-inositol biosynthesis ; myo-inositol from D-glucose 6-phosphate	step 2/2	cytoplasm	P29218
PLSP260	9606_human	Polyol metabolism ; myo-inositol biosynthesis ; myo-inositol from D-glucose 6-phosphate	step 2/2	membrane	Q9NX62
PLSP303	9606_human	Pyrimidine metabolism ; UMP biosynthesis via de novo pathway ; UMP from HCO(3)(-)	step 1/6	cytoplasm	P27708
PLSP303	9606_human	Pyrimidine metabolism ; UMP biosynthesis via de novo pathway ; UMP from HCO(3)(-)	step 2/6	cytoplasm	P27708
PLSP303	9606_human	Pyrimidine metabolism ; UMP biosynthesis via de novo pathway ; UMP from HCO(3)(-)	step 3/6	cytoplasm	P27708
PLSP303	9606_human	Pyrimidine metabolism ; UMP biosynthesis via de novo pathway ; UMP from HCO(3)(-)	step 4/6	mitochondria	Q02127
PLSP318	9606_human	Steroid biosynthesis ; zymosterol biosynthesis ; zymosterol from lanosterol	step 1/6	endoplasmic reticulum	Q16850
PLSP318	9606_human	Steroid biosynthesis ; zymosterol biosynthesis ; zymosterol from lanosterol	step 1/6	microsome	Q16850
PLSP318	9606_human	Steroid biosynthesis ; zymosterol biosynthesis ; zymosterol from lanosterol	step 3/6	endoplasmic reticulum	Q15800
PLSP318	9606_human	Steroid biosynthesis ; zymosterol biosynthesis ; zymosterol from lanosterol	step 4/6	membrane	Q15738
PLSP318	9606_human	Steroid biosynthesis ; zymosterol biosynthesis ; zymosterol from lanosterol	step 5/6	cell membrane	P56937
PLSP332	9606_human	Terpene metabolism ; lanosterol biosynthesis ; lanosterol from farnesyl-PP	step 1/3	endoplasmic reticulum	P37268
PLSP332	9606_human	Terpene metabolism ; lanosterol biosynthesis ; lanosterol from farnesyl-PP	step 2/3	endoplasmic reticulum	Q14534
PLSP332	9606_human	Terpene metabolism ; lanosterol biosynthesis ; lanosterol from farnesyl-PP	step 2/3	microsome	Q14534
PLSP85	9823_mammals	Carbohydrate degradation ; glycolysis ; pyruvate from D-glyceraldehyde 3-phosphate	step 1/5	cytoplasm	P00355
PLSP85	9823_mammals	Carbohydrate degradation ; glycolysis ; pyruvate from D-glyceraldehyde 3-phosphate	step 2/5	cytoplasm	Q7SIB7,Q6RI85
PLSP85	9823_mammals	Carbohydrate degradation ; glycolysis ; pyruvate from D-glyceraldehyde 3-phosphate	step 4/5	cytoplasm	Q1KYT0
PLSP85	9823_mammals	Carbohydrate degradation ; glycolysis ; pyruvate from D-glyceraldehyde 3-phosphate	step 4/5	myofibril	Q1KYT0
PLSP318	9823_mammals	Steroid biosynthesis ; zymosterol biosynthesis ; zymosterol from lanosterol	step 1/6	endoplasmic reticulum	O46420
PLSP318	9823_mammals	Steroid biosynthesis ; zymosterol biosynthesis ; zymosterol from lanosterol	step 1/6	microsome	O46420
PLSP318	9823_mammals	Steroid biosynthesis ; zymosterol biosynthesis ; zymosterol from lanosterol	step 3/6	endoplasmic reticulum	Q6UGB2
PLSP181	9913_mammals	Isoprenoid biosynthesis ; isopentenyl-PP biosynthesis via mevalonic acid pathway ; isopentenyl-PP from (R)-mevalonic acid	step 1/3	cytoplasm	Q5E9T8
PLSP181	9913_mammals	Isoprenoid biosynthesis ; isopentenyl-PP biosynthesis via mevalonic acid pathway ; isopentenyl-PP from (R)-mevalonic acid	step 1/3	peroxisome	Q5E9T8
PLSP181	9913_mammals	Isoprenoid biosynthesis ; isopentenyl-PP biosynthesis via mevalonic acid pathway ; isopentenyl-PP from (R)-mevalonic acid	step 2/3	peroxisome	Q2KIU2
PLSP231	9913_mammals	Phospholipid metabolism ; CDP-diacylglycerol biosynthesis ; CDP-diacylglycerol from sn-glycerol 3-phosphate	step 1/3	mitochondria	Q5GJ77

PLSP231	9913_mammals	Phospholipid metabolism ; CDP-diacylglycerol biosynthesis ; CDP-diacylglycerol from sn-glycerol 3-phosphate	step 2/3	membrane	Q95JH2,Q5E9R2,A3FPG8
PLSP231	9913_mammals	Phospholipid metabolism ; CDP-diacylglycerol biosynthesis ; CDP-diacylglycerol from sn-glycerol 3-phosphate	step 3/3	mitochondria	A0JNC1
PLSP260	9913_mammals	Polyol metabolism ; myo-inositol biosynthesis ; myo-inositol from D-glucose 6-phosphate	step 1/2	cytoplasm	Q2NL29
PLSP260	9913_mammals	Polyol metabolism ; myo-inositol biosynthesis ; myo-inositol from D-glucose 6-phosphate	step 2/2	cytoplasm	P20456
PLSP260	9913_mammals	Polyol metabolism ; myo-inositol biosynthesis ; myo-inositol from D-glucose 6-phosphate	step 2/2	membrane	Q2KJ53
PLSP85	9986_mammals	Carbohydrate degradation ; glycolysis ; pyruvate from D-glyceraldehyde 3-phosphate	step 1/5	cytoplasm	P46406
PLSP85	9986_mammals	Carbohydrate degradation ; glycolysis ; pyruvate from D-glyceraldehyde 3-phosphate	step 4/5	cytoplasm	P25704
PLSP85	9986_mammals	Carbohydrate degradation ; glycolysis ; pyruvate from D-glyceraldehyde 3-phosphate	step 4/5	myofibril	P25704
