# Supplementary material for: PathLocdb: a comprehensive database for the subcellular localization of metabolic pathways and its application to multiple localization analysis
Source: BMC Genomics. 2010 Dec 2;11(Suppl 4):S13. doi: 10.1186/1471-2164-11-S4-S13 (PMC3005916; doi:10.1186/1471-2164-11-S4-S13)
Supplement: Additional file 4 — The 45 genes from 17 of the 28 pathways which are detected to be duplicated during evolution are shown in Additional file 4. [file 1471-2164-11-S4-S13-S4.pdf]

Superpatwhay ID	Superpathway name	Location	UniProt accession number	Mapping to the gene duplication dataset from two published paper
PLSP82	Carbohydrate biosynthesis; gluconeogenesis	mitochondria	P11498	NM_000920
PLSP82	Carbohydrate biosynthesis; gluconeogenesis	mitochondria	P11498	NM_022172
PLSP94	Carbohydrate metabolism; hexose metabolism	mitochondria	P52789	NM_000189
PLSP96	Carbohydrate metabolism; tricarboxylic acid cycle	mitochondria	O14521	NM_003002
PLSP96	Carbohydrate metabolism; tricarboxylic acid cycle	mitochondria	O75390	NM_004077
PLSP96	Carbohydrate metabolism; tricarboxylic acid cycle	mitochondria	P31040	NM_004168
PLSP166	Glycan metabolism; heparan sulfate biosynthesis	endoplasmic reticulum	Q9UBQ6	NM_001439
PLSP166	Glycan metabolism; heparan sulfate biosynthesis	extracellular	Q9UBQ6	NM_001439
PLSP173	Glycolipid biosynthesis; glycosylphosphatidylinositol-anchor biosynthesis	endoplasmic reticulum	O95427	NM_012327
PLSP173	Glycolipid biosynthesis; glycosylphosphatidylinositol-anchor biosynthesis	endoplasmic reticulum	Q07326	NM_002643
PLSP173	Glycolipid biosynthesis; glycosylphosphatidylinositol-anchor biosynthesis	endoplasmic reticulum	Q86VD9	NM_025163
PLSP173	Glycolipid biosynthesis; glycosylphosphatidylinositol-anchor biosynthesis	endoplasmic reticulum	Q92535	NM_002642
PLSP187	Lipid metabolism; fatty acid beta-oxidation	mitochondria	P55084	NM_000183
PLSP188	Lipid metabolism; fatty acid biosynthesis	endoplasmic reticulum	Q53GQ0	NM_016142
PLSP189	Lipid metabolism; fatty acid metabolism	mitochondria	P42765	NM_006111
PLSP190	Lipid metabolism; glycerolipid metabolism	mitochondria	Q53H12	NM_018238
PLSP194	Lipid metabolism; leukotriene D4 biosynthesis	cell membrane	P36269	NM_004121
PLSP194	Lipid metabolism; leukotriene D4 biosynthesis	cytoplasm	P16050	NM_001140
PLSP202	Lipid metabolism; sphingolipid metabolism	endoplasmic reticulum	O15269	NM_006415
PLSP227	One-carbon metabolism; tetrahydrofolate pathway	cytoplasm	P34896	NM_004169
PLSP232	Phospholipid metabolism ; phosphatidylcholine biosynthesis ; phosphatidylcholine from phosphocholine	cytoplasm	P49585	NM_005017
PLSP283	Protein modification; protein glycosylation	cytoplasm	O15305	NM_000303
PLSP283	Protein modification; protein glycosylation	endoplasmic reticulum	Q5BKT4	NM_032834
PLSP283	Protein modification; protein glycosylation	endoplasmic reticulum	Q92685	NM_005787
PLSP283	Protein modification; protein glycosylation	endoplasmic reticulum	Q9BT22	NM_019109

PLSP283	Protein modification; protein glycosylation	endoplasmic reticulum	Q9BVK2	NM_024079
PLSP283	Protein modification; protein glycosylation	golgi apparatus	O94766	NM_012200
PLSP283	Protein modification; protein glycosylation	golgi apparatus	P21217	NM_000149
PLSP283	Protein modification; protein glycosylation	golgi apparatus	P51993	NM_000150
PLSP283	Protein modification; protein glycosylation	golgi apparatus	Q11128	NM_002034
PLSP283	Protein modification; protein glycosylation	golgi apparatus	Q9UJ37	NM_006456
PLSP286	Protein modification; protein sumoylation	cytoplasm	P49792	NM_006267
PLSP286	Protein modification; protein sumoylation	nucleus	P49792	NM_006267
PLSP286	Protein modification; protein sumoylation	nucleus	Q9UBE0	NM_005500
PLSP287	Protein modification; protein ubiquitination	cytoplasm	P40337	NM_000551
PLSP287	Protein modification; protein ubiquitination	cytoplasm	Q12829	NM_006822
PLSP287	Protein modification; protein ubiquitination	cytoplasm	Q15034	NM_014606
PLSP287	Protein modification; protein ubiquitination	cytoplasm	Q16531	NM_001923
PLSP287	Protein modification; protein ubiquitination	cytoplasm	Q5XPI4	NM_022064
PLSP287	Protein modification; protein ubiquitination	cytoplasm	Q7L622	NM_017769
PLSP287	Protein modification; protein ubiquitination	cytoplasm	Q96PU5	NM_015277
PLSP287	Protein modification; protein ubiquitination	cytoplasm	Q9HAU4	NM_022739
PLSP287	Protein modification; protein ubiquitination	cytoplasm	Q9UBS8	NM_004290
PLSP287	Protein modification; protein ubiquitination	cytoplasm	Q9Y4K3	NM_004620
PLSP287	Protein modification; protein ubiquitination	nucleus	P40337	NM_000551
PLSP287	Protein modification; protein ubiquitination	nucleus	Q05086	NM_000462
PLSP287	Protein modification; protein ubiquitination	nucleus	Q16531	NM_001923
PLSP287	Protein modification; protein ubiquitination	nucleus	Q7L622	NM_017769
PLSP287	Protein modification; protein ubiquitination	nucleus	Q86XK2	NM_025133
PLSP287	Protein modification; protein ubiquitination	nucleus	Q99496	NM_007212
PLSP287	Protein modification; protein ubiquitination	nucleus	Q9HAU4	NM_022739
PLSP287	Protein modification; protein ubiquitination	nucleus	Q9UBS8	NM_004290
PLSP287	Protein modification; protein ubiquitination	vesicle	Q15034	NM_014606
PLSP321	Steroid biosynthesis; estrogen biosynthesis	cytoplasm	P14061	NM_000413
PLSP321	Steroid biosynthesis; estrogen biosynthesis	endoplasmic reticulum	Q53GQ0	NM_016142

The two paper are as following:

Bailey, J.A., et al., Recent segmental duplications in the human genome. Science, 2002. 297(5583): p. 1003-7.

Bailey, J.A., et al., Segmental duplications: organization and impact within the current human genome project assembly. Genome Res, 2001. 11(6): p. 1005-17.
